# Generation and maintenance of apical rib-like actin fibers in epithelial support cells of the *Drosophila* eye

**DOI:** 10.1242/dev.204931

**Published:** 2026-01-08

**Authors:** Abhi Bhattarai, Emily W. McGhie, Joshua C. Woo, Srijana Niraula, Patrick Rosetti, Jaxon M. Kim, Ezekiel Popoola, Ruth I. Johnson

**Affiliations:** Department of Biology, Wesleyan University, Middletown, CT 06457, USA

**Keywords:** Actin cytoskeleton, Cell shape, *Drosophila* eye, Pseudostratified epithelium, Epithelial cell

## Abstract

Heterogeneity and complexity of cytoskeletal structures, and how these are regulated, is poorly understood. Here, we use cells of the *Drosophila* pupal eye as models to explore diversity in the actin cytoskeleton. We found that different F-actin structures emerge in primary, secondary and tertiary pigment cells as they mature. Primary cells became characterized by dense accumulations of F-actin that we termed apical ribs of actin fibers (ARAFs). The formins Diaphanous and Dishevelled Associated Activator of Morphogenesis are essential for generation of ARAFs, which are connected into a network by α-Actinin, the villin Quail, and spectrins, and linked to the apical membrane by Quail and spectrins. ARAFs are similar to stress fibers and connect to adherens junctions. Impairing ARAFs indicated that this network maintains cortical tension and is crucial for primary cells to achieve their characteristic shapes. Our evaluation of the three-dimensional shape of primary cells revealed that ARAFs are essential for the shape of the curved apical membrane. Hence, a toolkit of conserved actin regulatory proteins builds and maintains a network of apical stress fibers that governs the morphology of primary cells.

## Teaser

**Summary:** High-resolution confocal microscopy and 3D rendering, together with RNA interference, identify an F-actin network that shapes the apical region of cells of the *Drosophila* eye and uncovers a set of proteins that build and maintain the network.

## INTRODUCTION

Many tissues are characterized by collections of cells with cell type-specific 3D architectures. The organ of Corti of the mammalian cochlea, with its distinctly shaped hair and support cells, is one remarkable example (Lim, 1986). The mammalian intestine and airway epithelia similarly contain cells that can be easily identified because of their distinct morphologies (Davis and Wypych, 2021; Eenjes et al., 2022; Hewitt and Lloyd, 2021). The *Drosophila* eye, in which the different cell types acquire characteristic shapes during pupal development, is an invertebrate example and an experimental model in which mechanisms that determine cell-specific architectures can be interrogated.

**Fig. 1.**
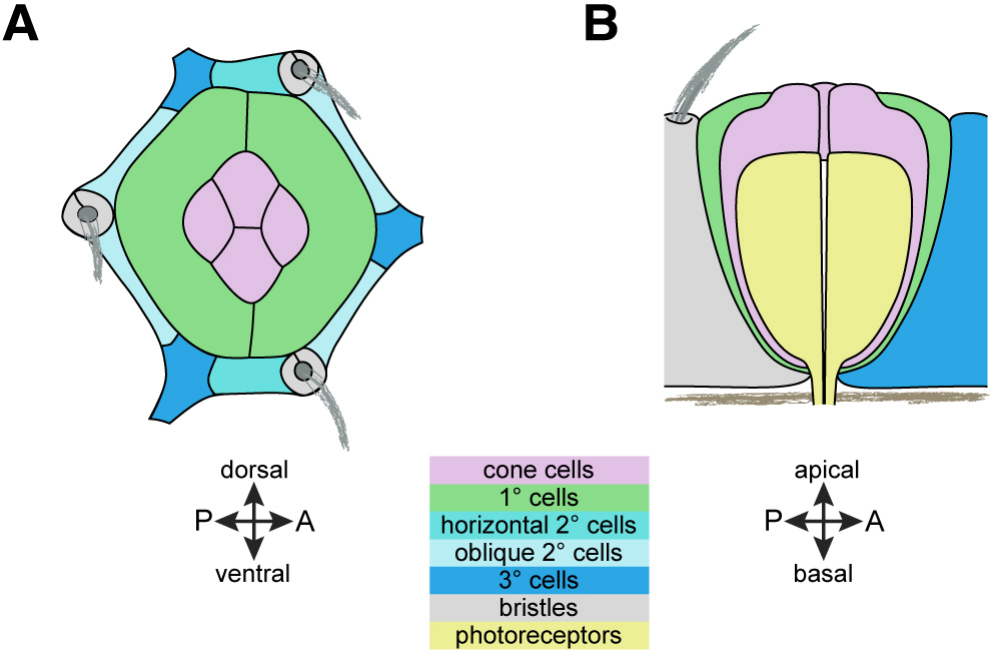
**The *Drosophila* ommatidium.** (A,B) Schematics of the apical face of an ommatidium at 40 h APF (A) and the ommatidium sliced longitudinally (B). Cell types are color-coded as shown in the key. A, anterior P, posterior.

The cells of the *Drosophila* compound eye emerge from a common lineage during larval and pupal development (Charlton-Perkins et al., 2021; Johnson, 2021; Kumar, 2012; Pichaud, 2014; Treisman, 2013). Eight photoreceptors form the core of each ommatidium of the eye. The eye has four types of epithelial support cells: the primary (1°), secondary (2°) and tertiary (3°) pigment cells; and cone cells (CCs) (Fig. 1). These epithelial cells enshroud the photoreceptor clusters at the core of each ommatidium to provide mechanical support, optically separate each ommatidium, and generate extracellular material that (apically) becomes organized into lenses that will cap each ommatidium, and (basally) contributes to an underlying fenestrated membrane. By 40 h after puparium formation (APF), successive morphogenetic processes have forged the epithelial cells into their final shapes, and detection of adherens junctions (AJs) reveals these cells as: two fabiform 1° cells that connect around the CCs of each ommatidium; CCs that organize like four adjoining soap bubbles; 2° cells that are rectangular with those placed horizontally between ommatidia wider than those positioned obliquely; and 3° cells that are roughly hexagonal. 2° and 3° cells are collectively called lattice cells (LCs).

The shapes that cells acquire are governed by many factors, including shape, density and stability of the cytoskeleton, actomyosin activity, junction composition and stability, placement of organelles, and forces transmitted from neighboring cells and the extracellular environment. Only a subset of these factors has been studied in the *Drosophila* eye (Johnson, 2021). Broadly, actin in epithelial cells is organized into a dynamic cortical network that supports the membrane, circumferential actin belts that connect to AJs in zonula adherens (ZA)-associated F-actin (ZA-actin), and basal stress fibers that connect via integrins to the extracellular matrix. When activated, myosin associates with these actin networks to introduce tension and drive cell and tissue shape change (Clarke and Martin, 2021; Quintanilla et al., 2023). In many developing tissues, pulsing of actomyosin activation is essential for junction remodeling, and repositioning and reshaping of cells (Balaghi and Fernandez-Gonzalez, 2024; Fernandez-Gonzalez and Harris, 2023; Miao and Blankenship, 2020). Similarly, in the *Drosophila* pupal eye, pulsatile activation of a pool of medial-apical actomyosin in LCs (from ∼26 h APF), and oscillations of contraction and growth of ZA-associated actomyosin at LC:LC junctions have been shown to be essential for development of the correct shape and size of LCs by 40 h APF (Del Signore et al., 2018; Malin et al., 2022, 2024; Rosa-Birriel et al., 2024). Pulsing of medial-apical actomyosin in 1° cells, and the accumulation of myosin at the CC:1° cell interface, has also been shown to be essential for the CCs to adopt their correct apical geometries (Blackie et al., 2021, 2020; Chan et al., 2017). These pupal eye studies have examined apical actomyosin during dynamic periods of pigment cell reorganization, pruning by apoptosis, and eye growth. How the cytoskeleton is organized to support the pigment cells once they have reached their final shapes has not yet been explored.

Strategies to visualize the actin cytoskeleton rely on introducing fluorescent molecules into the cells that bind actin filaments (F-actin). Tagged phalloidin remains the benchmark reagent for fixed preparations (Wulf et al., 1979), but for imaging F-actin in live tissues the standard strategy is to express fusion proteins of actin-binding domains (ABDs) linked to fluorescent proteins. ABDs used include the tail of *Drosophila* Moesin (Edwards et al., 1997), the calponin homology domain region of human utrophin (Burkel et al., 2007), a peptide derived from rat inositol 1,4,5-trisphosphate 3-kinase A termed F-tractin (Johnson and Schell, 2009), and a peptide of yeast Abp140 termed Lifeact (Riedl et al., 2008). These fusion proteins have been engineered as *Drosophila* transgenes (Bloor and Kiehart, 2001; Cai et al., 2014; Dutta et al., 2002; Edwards et al., 1997; Hatan et al., 2011; Huelsmann et al., 2013; Kiehart et al., 2000; Santa-Cruz Mateos et al., 2020; Spracklen et al., 2014; Zanet et al., 2012) and used to track cytoskeletal organization and dynamics in various *Drosophila* tissues.

*Drosophila* tissues have proven invaluable in revealing ways in which actin, and actomyosin complexes, are deployed to regulate cell behavior and drive development. We set out to characterize the actin cytoskeleton in epithelial cells of the *Drosophila* pupal eye with the hypothesis that F-actin would be employed differently to support the specific shape that each cell type acquires by 40 h APF. We found that the apical cortical cytoskeleton becomes organized into ‘starburst’ patterns in 3° cells and horizontal 2° cells, and is randomly organized in oblique 2° cells. In contrast, 1° cells become packed with an organized mass of actin cables that tracks across the cell width and that we termed apical ribs of actin fibers (ARAFs). These findings hence establish these cells as models for future work that will interrogate biophysical and genetic mechanisms that generate or respond to these cytoskeletal structures. To facilitate these studies in 1° cells, we characterized the set of actin regulators that generate, organize and maintain the ARAFs, including several that are expressed at higher levels in 1° cells than in LCs.

Most studies of pupal eye morphogenesis, including those described above, have considered patterning two-dimensionally and from the perspective of the apical eye surface. Recent studies have begun to correct this, exploring patterning of the basal foot processes of LCs and/or how this coordinates with apical events (Gazso-Gerhat et al., 2023; Ready and Chang, 2023; Sun et al., 2024; Walther et al., 2024). Two of these studies present 3D renderings of 2° and 3° cells at ∼40 h APF (Gazso-Gerhat et al., 2023; Walther et al., 2024). We now present 3D renderings of 1° cells, which will correct misconceptions of how these cells are shaped. Our 3D analyses of the morphology of 1° cells and the ARAF network revealed that ARAFs maintain the elevated domed shape of ommatidia. Further, we find that ARAFs connect to AJs, the ZA are tensile, and are essential for the fabiform shape of 1° cells. This study therefore provides the first 3D analyses of 1° cells and the cytoskeletal network that scaffolds the extraordinary apical shape of this cell type, thus providing a conceptual framework for the genetic control of a tensile cytoskeletal network that is essential for a complex cell shape to emerge in an epithelium.

## RESULTS

### Cell-specific apical-cortical actin structures form in the *Drosophila* pupal eye

**Fig. 2.**
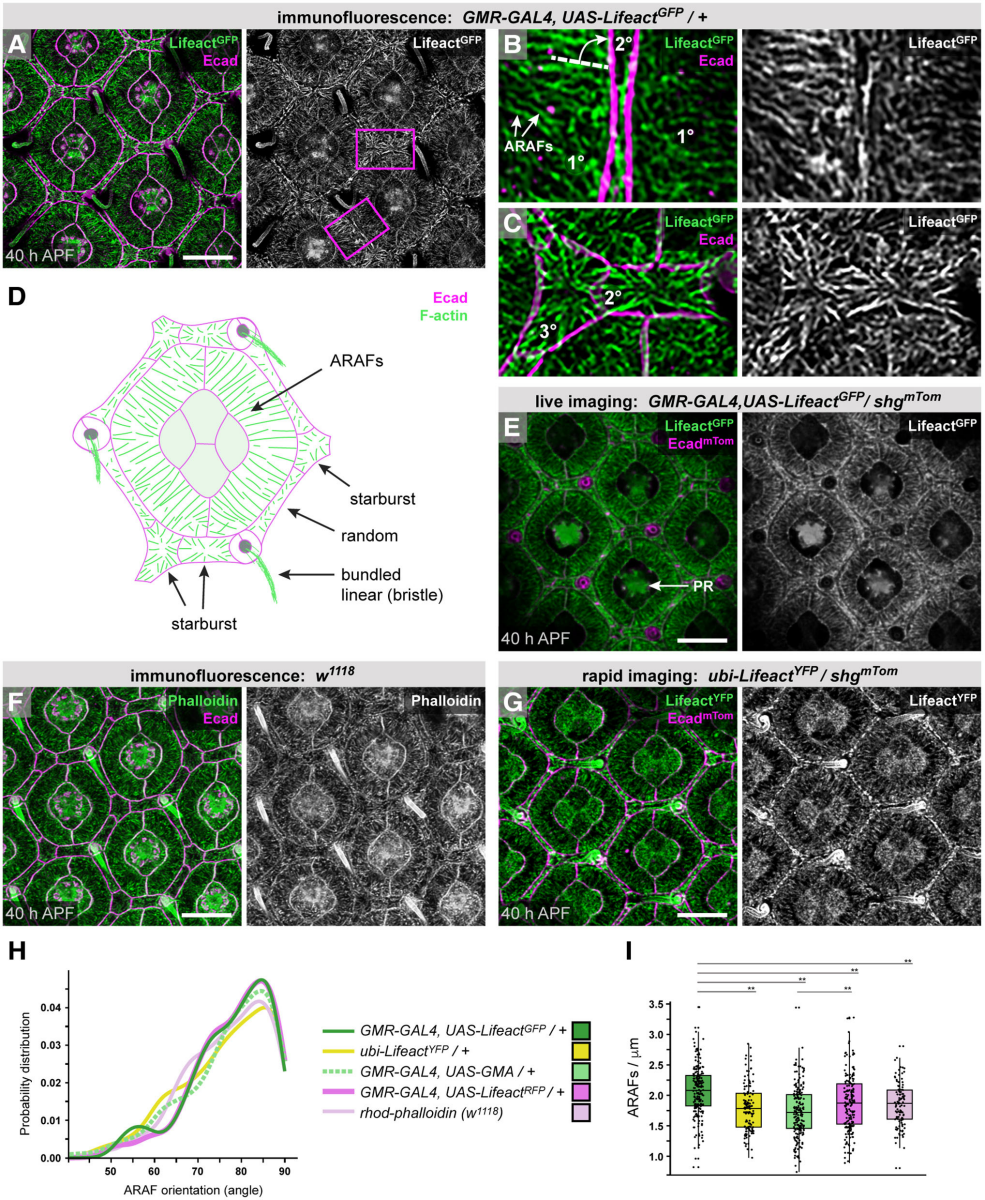
**Cell type-specific actin structures form in epithelial support cells of the eye.** (A-C) Small region of an eye (A), with *Lifeact^GFP^* driven by *GMR-GAL4* and AJs marked by Ecad. Boxed regions are presented at higher magnification in B and C. In B, white arrows indicate two almost-parallel ARAFs, and white dashed line drawn at 84° to AJ indicates mean orientation of ARAFs with respect to the 1°:LC border. (D) Schematic summarizing apical-cortical F-actin organization in an ommatidium. Actin in CCs and at ZAs is excluded for simplicity. (E) Small region of the eye of a live pupa, with *Lifeact^GFP^* and endogenous Ecad^mTom^ (encoded by *shg^mTom^*). Lifeact^GFP^-rich photoreceptor rhabdomeres (PR) indicated with white arrow. (F) Ommatidia of a *w^1118^* eye, with F-actin detected with phalloidin. (G) Region of an eye with *ubi-Lifeact^YFP^* and Ecad^mTom^. (H) Distribution plot of the angle at which ARAFs oriented toward the 1°:LC boundary. (I) Analyses of the density of ARAFs in 1° cells; the box plot indicates the median and quartiles. **P<0.001. The images shown in A and F are also shown in Figs S1 and S4. See Tables S1 and S2 for statistical analyses and sample numbers for H and I. Scale bars: 10 µm.

Previous detection of F-actin with phalloidin suggested that the cytoskeleton becomes increasingly complex in epithelial cells of the pupal eye as they adopt their characteristic shapes (Johnson et al., 2008). We drove *UAS-Lifeact^GFP^* with *GMR-GAL4*, and coupled this approach with high-resolution confocal microscopy, to resolve in detail how F-actin becomes organized in these cells (Fig. 2, Fig. S1).

Our approach revealed numerous apical-cortical actin filaments, organized across the width of the fabiform 1° cells (Fig. 2A-G). We name these ARAFs to describe their morphology. Most ARAFs were oriented toward the boundary between 1° and 2° or 3° cells (LCs) at ∼84° (Fig. 2H). In hexagonal 3° cells apical-medial F-actin was organized with filaments tracking toward the approximate cell center in a ‘starburst’ formation (Fig. 2C,D). Similar starburst F-actin formation was observed in 2° cells positioned horizontally about each ommatidium (Fig. 2C,D), and F-actin was randomly oriented in the narrower oblique 2° cells (Fig. 2A,D). *GMR-GAL4* did not consistently drive *UAS-Lifeact^GFP^* in CCs, which we consequently excluded from further analyses.

Apical actin structures were fragile, so we developed strategies to prevent collapse or fragmentation of the cytoskeleton (see Materials and Methods). We were concerned that fixation would modify cytoskeletal organization, but we observed the same F-actin structures in retinas of live pupae (Fig. 2D) although image resolution was lower as the eye is surrounded by hemolymph and internal to the head with an overlying layer of epithelium through which one must focus. Lifeact may also introduce artifacts to the cytoskeleton, but F-actin appeared the same when detected with phalloidin (Fig. 2F), in retinas with low levels of Lifeact (driven with the *ubiquitin* (*Ubi-p63E*) promoter or *sparkling-GAL4*; Fig. 2G, Fig. S2A), and in retinas expressing the GFP-tagged ABD of Moesin (GMA; Fig. S2B,C). Whilst more ZA-actin was detected with phalloidin than with Lifeact (compare Fig. 2F with 2A), analyses of ARAFs labeled with Lifeact^GFP^, Lifeact^YFP^, Lifeact^RFP^, GMA or phalloidin confirmed that ARAFs were identically oriented regardless of the detection strategy (Fig. 2H), although ARAF density differed modestly (Fig. 2I). These minor disparities could be due to differences in emission or photostability of the fluorophores: Lifeact^YFP^ rapidly photobleached whilst Lifeact^GFP^ remained robust; rhodamine-phalloidin and Lifeact^RFP^ were less bright than Lifeact^GFP^ (Fig. 2I). Hence, we consider it unlikely that Lifeact significantly altered ARAF architecture.

### Formation of ARAFs correlates with rounding of the 1° cell:LC border

**Fig. 3.**
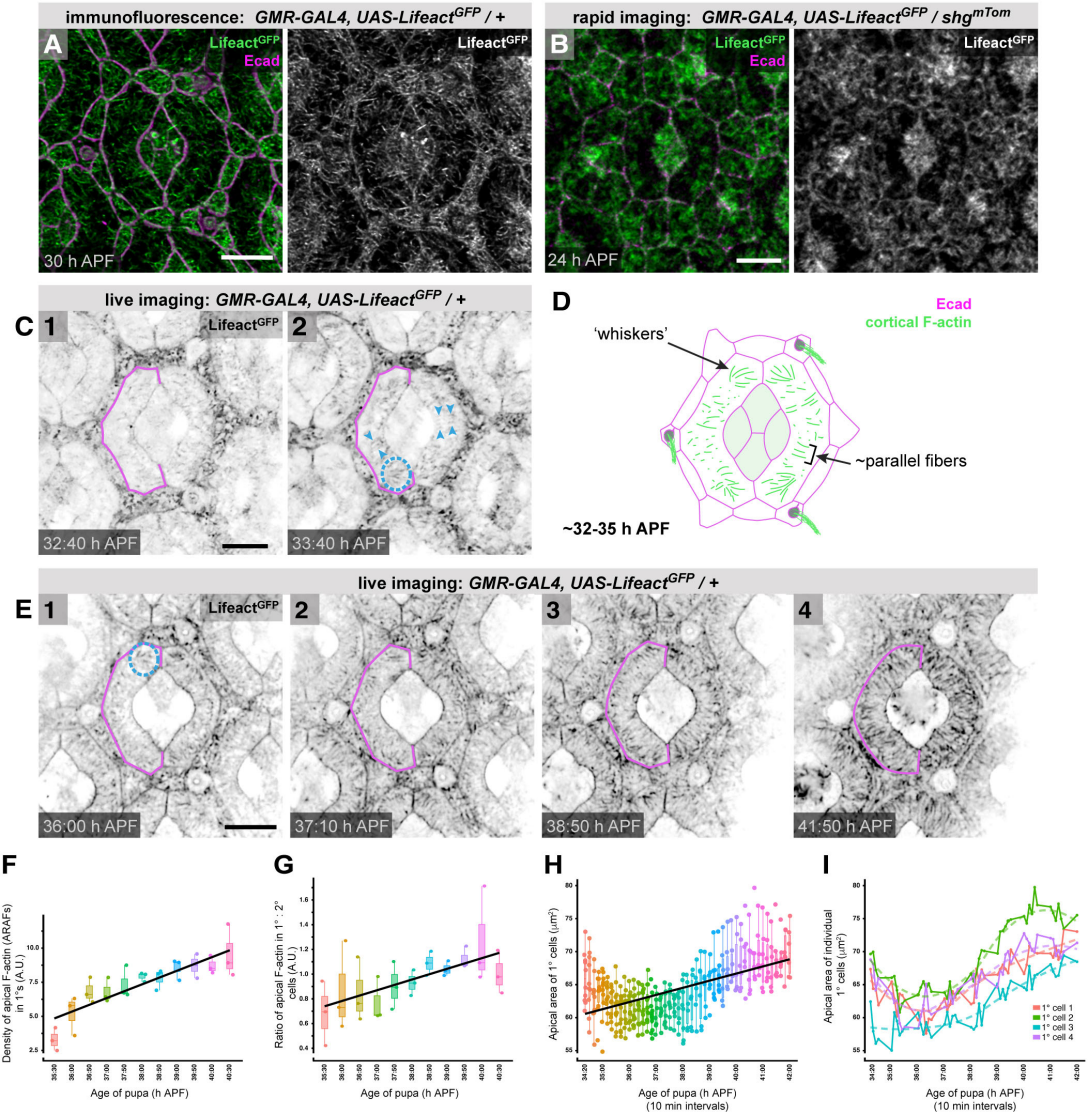
**The ARAF network emerges from ∼32 h APF.** (A,B) Lifeact^GFP^ and Ecad in an ommatidium at 30 h APF (A) and 24 h APF (B). (C) Stills (1 and 2) from an eye with Lifeact^GFP^, imaged live. Age of eye as indicated; see Fig. S3 for these and additional stills. Blue arrowheads indicate emergent ARAFs oriented in parallel. Dashed circle outlines a tuft of actin. (D) Illustration summarizing apical-cortical F-actin in 1° cells as ARAFs first appear. ZA-actin is excluded. (E) Stills (1-4) from Movie 1 of a live retina with Lifeact^GFP^. In C and E a 1° cell is outlined in magenta. (F-H) Analyses of 1° cells imaged live: (F) density of apical-cortical F-actin and emergent ARAF network, (G) ratio of F-actin in 1° and 2° cells, (H) apical area of 1° cells. Box plots indicate the median and quartiles. (I) Plot of the area of four individual 1° cells, imaged live. Dashed lines indicate trend lines. Scale bars: 5 µm. A.U., arbitrary units.

Given the extraordinary organization of ARAFs, we next explored when these were generated. Short randomly oriented actin filaments were observed filling the apical-medial domain of 1° cells from ∼30 h APF (Fig. 3A). Before then, a sparser, patchy meshwork of apical-medial F-actin was observed (Fig. 3B). Live imaging of retinas revealed that as longer filaments emerged in 1° cells a subset oriented roughly perpendicularly to the 1°:LC border and in parallel to each other (arrowheads in Fig. 3C, illustrated in Fig. 3D, Fig. S3A). In dorsal and ventral regions of many 1° cells, filaments were arranged in ‘whisker-like’ conformations (Fig. 3D, dashed circles in Fig. 3C2, E1) that became less prominent as the stereotypical ARAF network emerged (Fig. 3E2-E4). The nature of these actin whiskers is unclear. The density of ARAFs increased rapidly after 33 h APF as long fibers emerged that became oriented mainly across the width of 1° cells (Movie 1, Fig. 3E-G, Fig. S3). As at 40 h APF, similar actin conformations were observed in younger retinas, regardless of the method of F-actin detection (Fig. S4). Whether ARAFs were generated from reorganization of extant F-actin or instead generated *de novo* remains to be investigated.

**Fig. 4.**
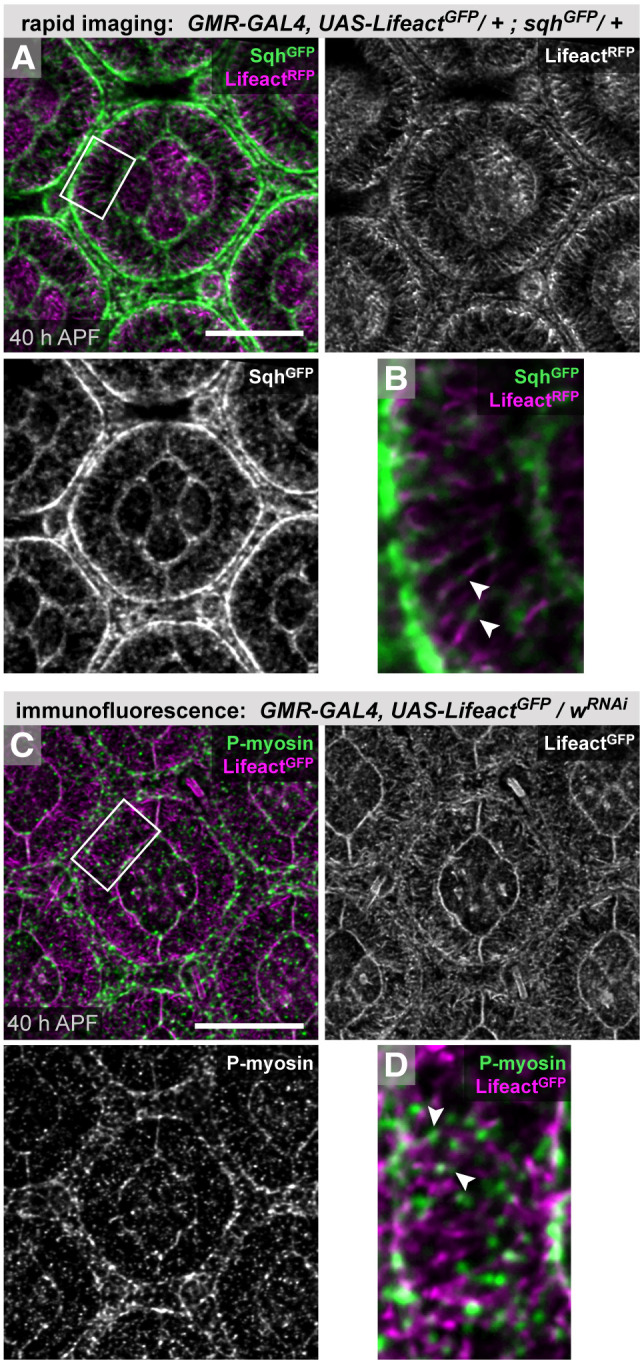
**NMII localizes to ARAFs.** (A,B) Lifeact^RFP^ and Sqh^GFP^ (a proxy for NMII) in an ommatidium at 40 h APF. Boxed region is shown at higher magnification in B, with white arrowheads indicating Sqh^GFP^ along ARAFs. Sqh^GFP^ also accumulated toward 1°:LC borders. (C,D) Immunofluorescence for phosphorylated myosin, with boxed region shown at higher magnification in D. Scale bars: 10 µm.

Scalloping of the boundaries between 1° cells and LCs was pronounced before 33-34 h APF (1° cells outlined in Fig. 3C, Fig. S3A) and ARAF emergence coincided with gradual relief of scalloping and adoption of the characteristic rounded fabiform of 1° cells (Fig. 3E), suggesting that ARAFs support adoption of this shape, although it is plausible that ARAFs emerge in response, instead, to rounding of 1° cells. Analyses of the 1° cell fabiforms showed an overall increase in area as the ARAF network assembled (Fig. 3H). However, this area oscillated within individual cells over time (Fig. 3I). Similar fluctuations in 1° cell area have been reported in younger retinas (well before ARAF emergence) and ascribed to oscillatory activity of actomyosin meshworks (Blackie et al., 2020). Accordingly, we detected spaghetti squash (Sqh), the regulatory light chain of non-muscle myosin II (NMII), and active phosphorylated NMII, along and between ARAFs (Fig. 4), suggesting that the ARAF network is tensile, with NMII activity driving oscillations in 1° cell size.

### Formation or maintenance of ARAFs is dependent on formins and Enabled

**Fig. 5.**
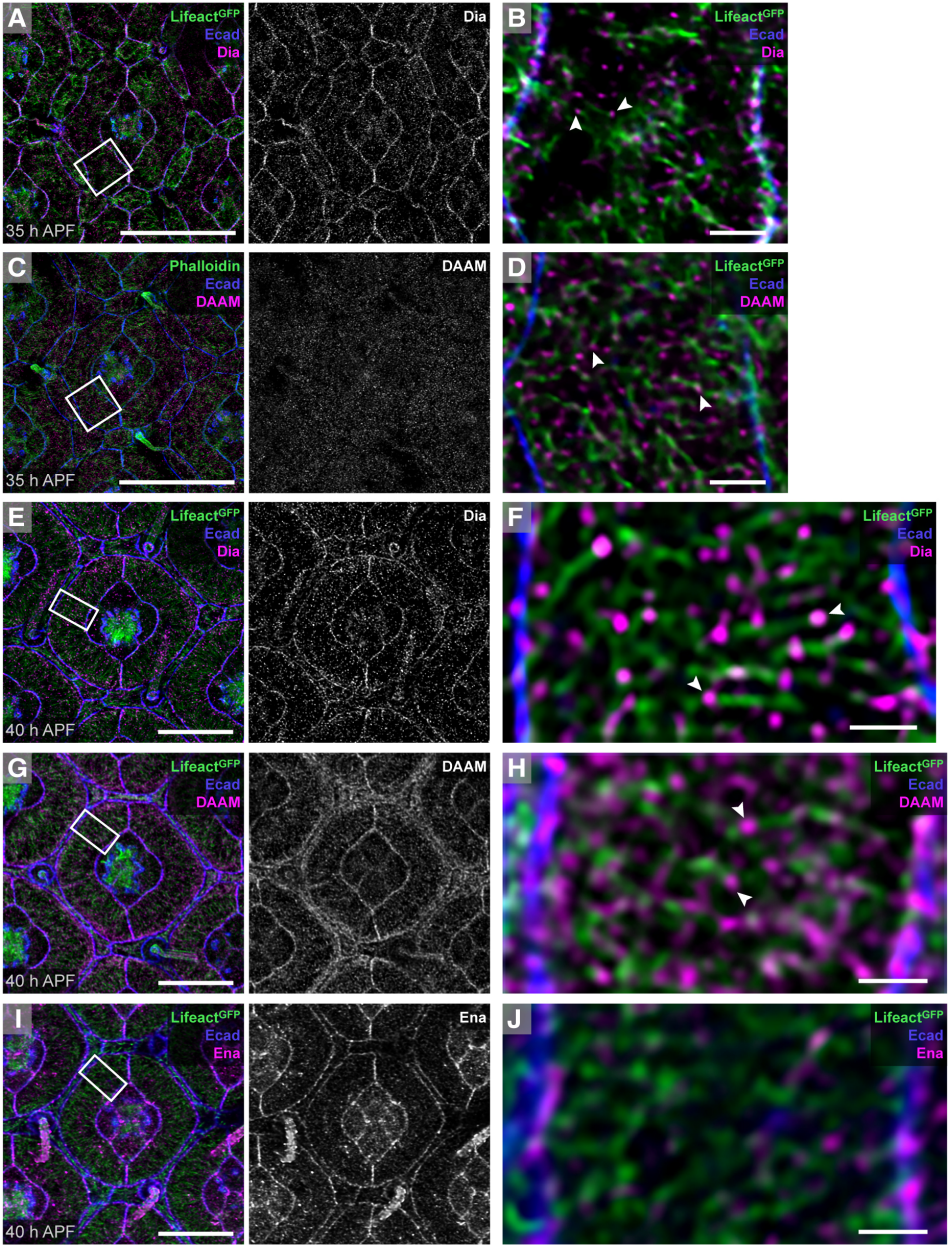
**DAAM, Dia and Ena localize to ARAFs and/or AJs.** (A,B) Dia in an ommatidium at 35 h APF, with boxed region shown at higher magnification in B. (C,D) DAAM in 35 h APF ommatidia, with boxed region enlarged in D. (E-H) Dia (E) and DAAM (G) in ommatidia at 40 h APF, with boxed regions shown at higher magnification in F and H at high magnification. White arrowheads in B, D, F and H point to Dia or DAAM at the tips of ARAFs. (I,J) Ena in an ommatidium at 40 h APF, with boxed region shown at higher magnification in J. Scale bars: 1 µm (B,D,F,H,J); 10 µm (A,C,E,G,I).

We predicted that formins were instrumental in generating ARAFs and our survey of the *Drosophila* formins Cappuccino (Capu) (Emmons et al., 1995; Quinlan et al., 2005; Rosales-Nieves et al., 2006), Diaphanous (Dia) (Afshar et al., 2000; Castrillon and Wasserman, 1994; Grosshans et al., 2005), Dishevelled Associated Activator of Morphogenesis (DAAM) (Matusek et al., 2006, 2008), Formin 3 (Form3) (Tanaka et al., 2004), Formin homology 2 domain containing (Fhos) (Anhezini et al., 2012; Lammel et al., 2014) and Formin-like (Frl) (Bai et al., 2011; Rohn et al., 2011) revealed that DAAM and Dia were present in the pupal eye before and during ARAF generation (Fig. 5A-H, Fig. S5A,B). DAAM and Dia are activated by Rho GTPases (Kühn and Geyer, 2014), and, consistent with this, a Rho/Rac activity sensor localized to the cortical region of 1° cells during ARAF assembly (Fig. S5C) (Abreu-Blanco et al., 2014).

Formins attach to and extend the barbed ends of F-actin (Breitsprecher and Goode, 2013; Courtemanche, 2018). Dia and DAAM localized to the tips of many actin filaments oriented across the 1° cell width at 35 h APF (Fig. 5B,D) and 40 h APF (Fig. 5F,H), indicating that the ARAF network is composed of filaments that tile across the 1° cell width. Dia also accumulated at AJs between 1° cells and their neighbors (Fig. 5A,E), consistent with the role of Dia family formins in supporting ZA-actin and AJ stability (Carramusa et al., 2007; Homem and Peifer, 2008; Sahai and Marshall, 2002). In contrast, DAAM accumulated at AJs by 40 h APF but was absent from junctions at 35 h APF (Fig. 5C,G). Another barbed-end actin polymerizer, Enabled (Ena; a *Drosophila* vasodilator-stimulated phosphoprotein) (Gertler et al., 1995; Winkelman et al., 2014), localized to AJs throughout the retina (Fig. 5I,J), consistent with AJ localization of Ena in other tissues (Faix and Rottner, 2022).

**Fig. 6.**
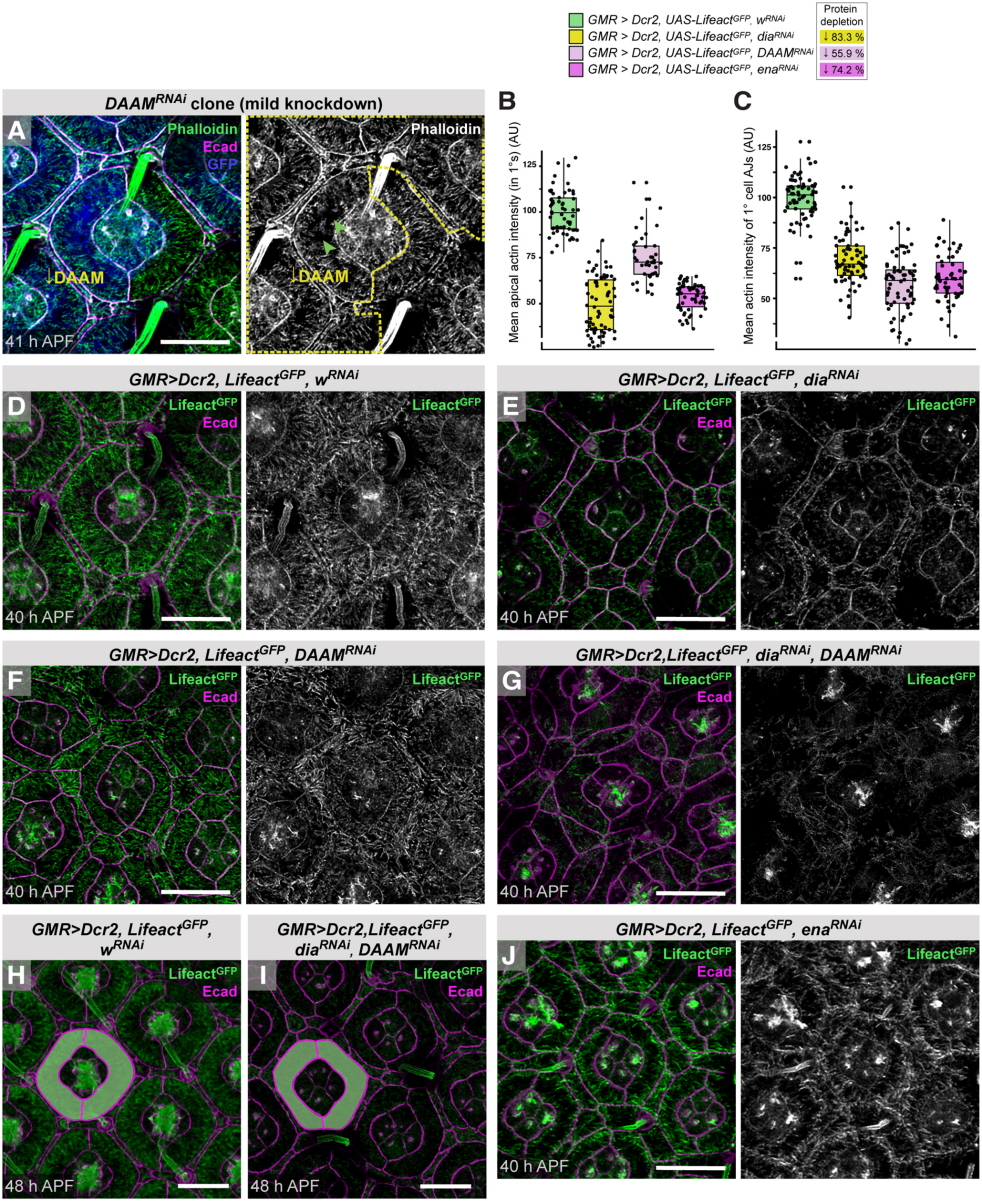
**ARAFs are generated and maintained by formins.** (A) Clone of *DAAM^RNAi^* expression labeled with GFP (blue). The left 1° cell but not right one has *DAAM^RNAi^*. Arrowheads highlight gaps in F-actin (phalloidin). Dashed yellow line outlines cells with *DAAM^RNAi^*. (B,C) Analyses of the intensity of apical-cortical Lifeact^GFP^ in 1° cells as a proxy for ARAF density (B) and Lifeact^GFP^ at ZA-actin at 1°:LC junctions (C) in the indicated genotypes. Box plots indicate medians and quartiles; sample numbers and statistical analyses are summarized in Tables S4 and S5. (D-J) Control ommatidia with *w^RNAi^* expression (D), and ommatidia with *dia^RNAi^* (E), *DAAM^RNAi^* (F), both *dia^RNAi^* and *DAAM^RNAi^* (G,I), *w^RNAi^* (H) and *ena^RNAi^* (J) expression. Ecad labels AJs and F-actin detected by co-expression of *Lifeact^GFP^*. 1° cells in H and I are outlined to highlight cell shape. Scale bars: 10 µm. AU, arbitrary units.

To test the contributions of Dia and DAAM to ARAF formation, we next reduced their translation using RNA interference (RNAi). Clones of retinal cells expressing RNAi transgenes against *dia*, *DAAM* or both loci were cell lethal or severely disrupted eye patterning, unless generated late or using mild transgenes, which failed to reduce protein expression significantly and only modestly disrupted ARAFs (Fig. 6A, left 1° cell has *DAAM^RNAi^*, green arrowheads highlight gaps in the ARAF network). Further, the *spa-GAL4* line, which drives expression in 1° cells and CCs (Fig. S2A) (Fu and Noll, 1997), drove transgene expression too weakly to disrupt target proteins. Hence, we drove RNAi expression across the eye using *GMR-GAL4*, which in our hands seldom impacted protein expression during larval or early pupal development (leaving earlier development unimpeded) and sufficiently reduced protein levels by the time of ARAF formation (Table S3, Fig. S6). However, this affected protein expression throughout the eye and introduced defects also into LCs. This approach severely reduced Dia (by 83.3% by 40 h APF; Table S3) and disrupted the ARAF network: only sparse short fragments of F-actin were observed in 1° cells by 40 h APF (Fig. 6B-E). *DAAM^RNAi^* transgenes reduced DAAM less efficiently (by 55.9%; Table S3), but ARAFs were nonetheless short, less dense, and randomly oriented (Fig. 6B,F). Reducing both Dia and DAAM simultaneously impeded ARAFs most severely (Fig. 6G).

Reducing formin expression also severely reduced ZA-actin (Fig. 6C-G). This reduction was, surprisingly, more significant in retinas with reduced DAAM than those with reduced Dia (Fig. 6C) indicating that, despite being absent from AJs in younger retinas, DAAM has a more prominent role than Dia in generating ZA-actin in 1° cells. Indeed, many 1° cell pairs failed to fully encircle the CCs and securely adhere to each other when DAAM was reduced (Fig. 6F), suggesting that DAAM is particularly crucial in securing adhesion between 1° cells.

1° cells were seldom correctly rounded and had reduced apical areas when Dia and/or DAAM were reduced across the eye (Fig. 6E-G), but these errors were likely consequent to defects in adjoining LCs which were larger than in control retinas or remained rounded (compare to LCs in Fig. 6D). By 48 h APF, however, LCs had narrowed and mainly organized into a hexagonal lattice and 1° cells became rounded, but 1° cell width was still less regular than in control retinas and the fabiform shape mildly distorted (Fig. 6H,I). These data suggest that ARAFs contribute to the uniform shape of 1° cells. Indeed, when we lowered formin activity in one 1° cell of an ommatidium to modestly disrupt ARAFs, that 1° cell was marginally larger, indicating that the ARAF network limits the width of 1° cells (Fig. 6A, left 1° cell is marginally larger than right 1° cell, Fig. S7). These data are consistent with the ARAF network being contractile (Fig. 4). Accordingly, ablating ARAFs in *GMR>dia^RNAi^* or *GMR>DAAM^RNAi^* retinas left little NMII activity in 1° cells, although 1° cells were crowded by larger LCs in these genotypes and consequently smaller (Fig. S8).

Despite not localizing to ARAFs, reducing Ena (by 74.2%; Table S3) reduced the density of ARAFs, but many that remained still traversed much of the 1° cell width (Fig. 6B,J, Table S4). ZA-actin was, however, severely reduced in 1° cells (Fig. 5C,J), consistent with role for Ena/VASP family proteins in assembly or maintenance of ZA-actin (Baum and Perrimon, 2001; Leerberg et al., 2014; Scott et al., 2006; Yu-Kemp et al., 2017). Hence, we posit that Ena does not contribute to ARAF elongation but instead to generation of ZA-actin, which is required for formation or maintenance of a dense ARAF network. This implies that the ARAF network interacts with ZA-actin.

### The ARAF network connects to the zonula adherens

**Fig. 7.**
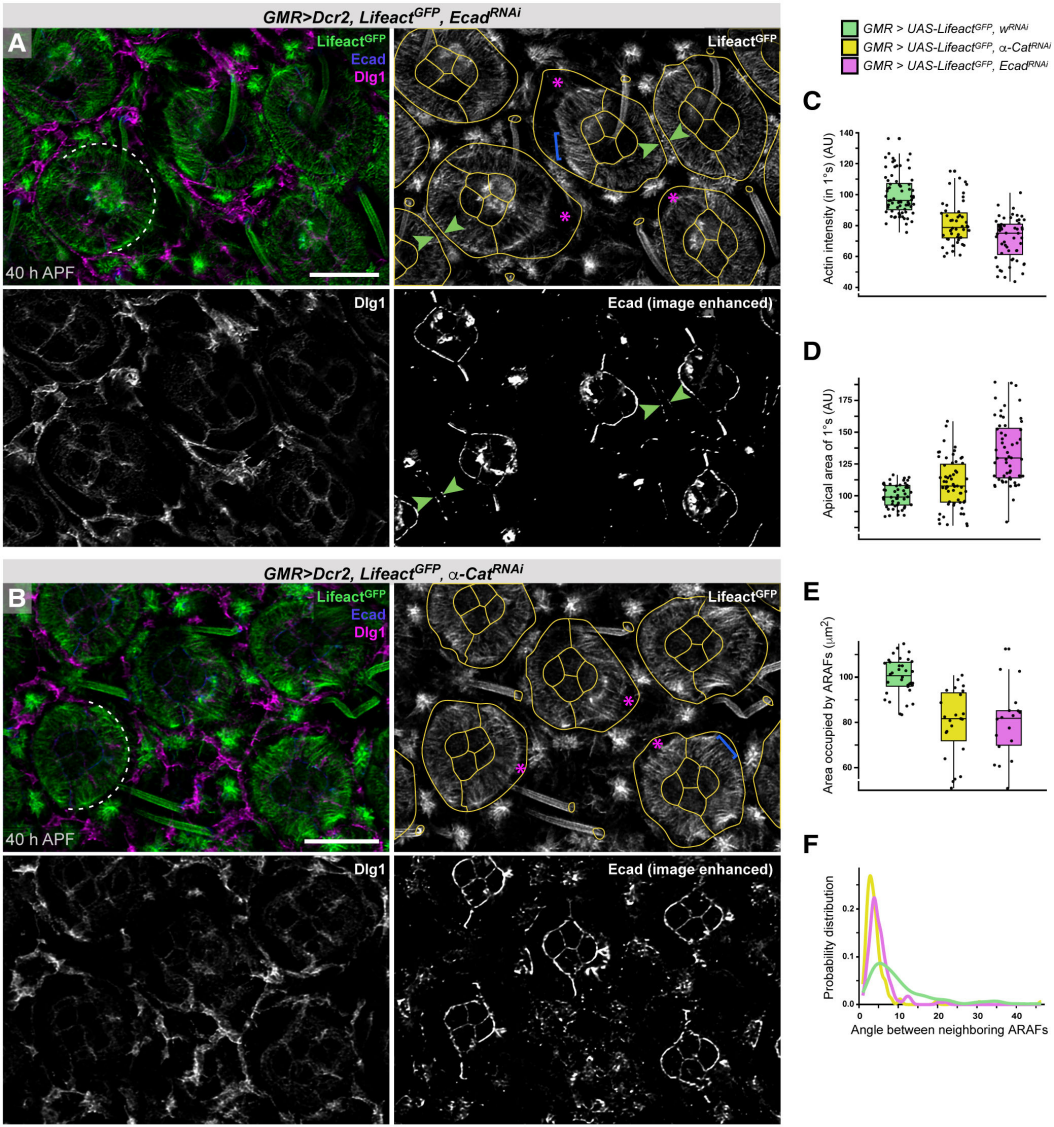
**The ARAF network connects to the zonula adherens.** (A,B) Small region of an eye with *Ecad^RNAi^* (A) or *α-Cat^RNAi^* (B). See Fig. S8 for control ommatidium with *w^RNAi^* expression. Lifeact^GFP^ labels F-actin, Ecad labels AJs, and Dlg1 marks lateral cell membranes. Dashed white semi-circle annotates the shape of an ARAF network released from the 1°:LC boundary. Ommatidia and bristle collars are traced in yellow. Magenta asterisks indicate examples where ARAFs are disconnected from the 1°:LC boundary. Blue brackets indicate released almost-parallel ARAFs. Green arrowheads indicate ARAFs still connected to remaining AJs. The images in A and B are also shown in Fig. S9. (C-F) Analyses of the intensity of apical-cortical actin in 1° cells (C), the area occupied by 1° cells (D) or the ARAF network (E), and the distribution of the orientation of neighboring ARAFs (F). Box plots indicate medians and quartiles; sample numbers and statistical analyses are summarized in Tables S6-S9. Scale bars: 10 µm. AU, arbitrary units.

To test our hypothesis that ARAFs synapse with the ZA, we directly disabled ZA junctions (ZAJs) by reducing E-cadherin (Ecad, encoded by *shotgun* in *Drosophila*) or α-Catenin (α-Cat, which links Ecad to F-actin) (Mege and Ishiyama, 2017). Driving RNAi transgenes with *GMR-GAL4* generated ommatidia with sparse AJs between 1° cells and their LC neighbors but AJs at 1°:CC boundaries were less disrupted (Fig. 7); *spa-GAL4* restricted transgene expression to 1° cells but failed to sufficiently drive transgenes to disrupt AJs.

Although actin density was only modestly reduced (Fig. 7C, Fig. S9A), the ARAF network was profoundly modified when the outer 1°:LC AJs were disrupted. First, gaps (asterisks in Fig. 7A,B) emerged between ARAFs and 1°:LC boundaries (detected via membrane-associated Discs large, Dlg1) and 1° cells bulged into the surrounding tissue at these gaps, becoming enlarged at their apical areas (Fig. 7D, cell outlines traced in Fig. 7A,B). In 1° cells in which the ARAF network was entirely disconnected from the outer 1°:LC border, the network was shaped into an almost-perfect half-ring that echoed the rounded fabiform of mature 1° cells (dashed semi-circles in Fig. 7A,B), and the area occupied by ARAFs was reduced (Fig. 7E). These data suggest that ARAFs are usually stretched across 1° cells and constrain 1° cells into their fabiform shape. Remarkably, the presence of just a few AJs along the outer 1°:LC boundary was sufficient to tether the ARAF network and constrain the 1° cell width (Fig. 7A, arrowheads).

Second, disconnecting ARAFs from AJs the ZA altered organization of the network. In control retinas, most ARAFs were oriented at angles of ∼6° between neighboring fibers so that they ‘fanned’ toward the outer 1°:LC boundary but disrupting AJs significantly reduced fanning of ARAFs (Fig. 7F, Fig. S9A) and groups of ARAFs were observed organized approximately in parallel (Fig. 7A,B, blue brackets). Hence, connecting the ARAF network to the ZA is essential to fan ARAFs appropriately.

Reducing Ecad or α-Cat also generated striking cytoskeletal defects in LCs. Apical F-actin was absent from LCs in which *Ecad^RNAi^* obliterated all AJs, but in LCs with remnant AJs tufts of F-actin were observed, usually adjacent to remaining AJs (Fig. S9B,C). In LCs with *α-Cat^RNAi^*, actin radiated from aggregates of cytoplasmic Ecad puncta that we predict are endocytic vesicles of destabilized Ecad (Fig. S9B,C). These data suggest that AJs scaffold actin nucleators or regulators in LCs, as has been established in other systems (for example, Grikscheit et al., 2015; Kobielak et al., 2004; Kovacs et al., 2002, 2011; Magie et al., 2002; Sousa et al., 2005; Tang and Brieher, 2012).

### ARAFs are crosslinked into a network and connected to the apical plasma membrane

We next interrogated the relationship between ARAFs and the apical plasma membrane. Spectrin complexes link F-actin to the cell membrane and, in also crosslinking actin filaments, can introduce order into a cortical actin network (Leterrier and Pullarkat, 2022; Lorenzo et al., 2023). For example, in axons, cortical rings of actin are tethered to the membrane, linked to each other, and periodically spaced by spectrins. We hypothesized that spectrins might similarly organize and anchor ARAFs.

**Fig. 8.**
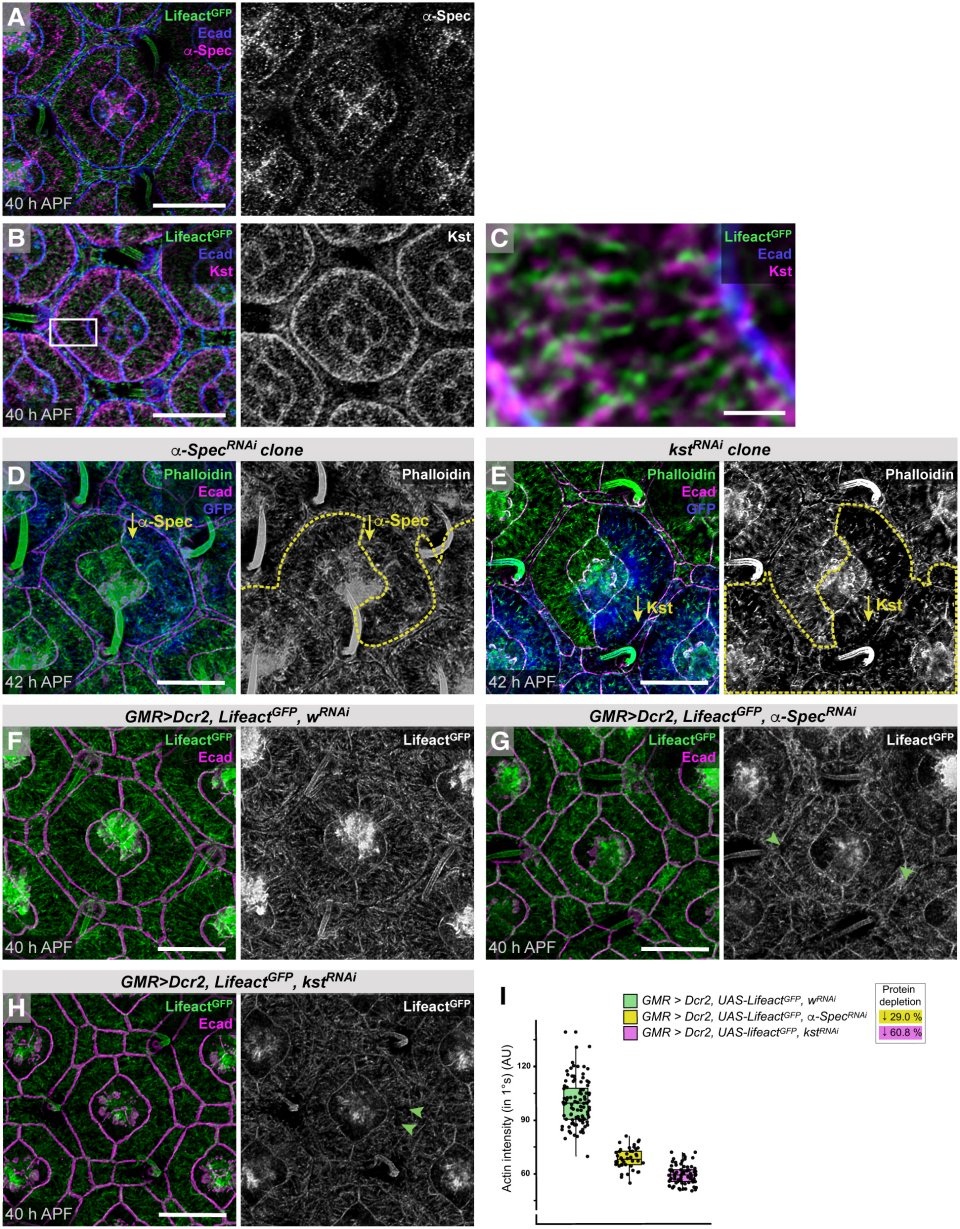
**Spectrins connect ARAFs to the cell membrane.** (A-C) α-Spec (A) and Kst (B) in ommatidia at 40 h APF. Lifeact labels F-actin and Ecad marks AJs. The boxed region in B is shown at higher magnification in C. (D,E) Hybrid ommatidia with the right 1° cell expressing α-*Spec^RNAi^* (D) or *kst^RNA^* (E). Phalloidin labels F-actin and yellow dashed lines outline tissue with RNAi transgene expression, marked by GFP (in blue). (F-H) Control ommatidium with *w^RNAi^* expression (F) and ommatidia with α-*Spec^RNAi^* (G) or *kst^RNAi^* (H). Green arrowheads indicate gaps in the ARAF network. (I) Quantification of the intensity of apical-cortical F-actin; box plots indicate medians and quartiles; sample numbers and statistical analyses are summarized in Table S10. Scale bars: 1 µm (C); 10 µm (A,B,D-H). AU, arbitrary units.

Spectrins are tetramers, composed of two heterodimers, which in *Drosophila* consist of α-Spectrin (α-Spec) and β-Spectrin (β-Spec) or β_H_-Spectrin (encoded by *karst*, *kst*) (Byers et al., 1992; Deng et al., 1995; Dubreuil et al., 1987, 1990). We detected α-Spec and β-Spec at apical and basolateral membranes of all pupal eye cells whilst Kst was enriched at apical membranes and expressed at higher levels in 1° cells than in LCs, and localized along, adjacent to and between ARAFs (Fig. 8A-C). Similar spectrin distributions have been reported previously in *Drosophila* eye and other cells (Lee et al., 2010, 1997; Thomas and Kiehart, 1994; Thomas and Williams, 1999; Thomas et al., 1998). These data suggested that α-Spec/Kst complexes link ARAFs and tether the network to the apical membrane. To test this, we generated hybrid ommatidia with one 1° cell expressing *kst^RNAi^* or α-*Spec^RNAi^*. ARAFs were disrupted in these 1° cells, which were not uniformly fabiform and were consistently larger than wild-type partner 1° cells (Fig. 8D,E, Fig. S7). This increase in cell area concurs with an earlier description of spectrins in maintaining cortical tension to restrict the cell size in the pupal eye (Deng et al., 2020). Our data now show that in 1° cells it is the contractile ARAF network that introduces cortical tension to restrict apical area.

When we reduced *α-Spec* or *kst* expression more severely and across the eye (by 29.03% and 60.8%, respectively; Table S3), ARAF network density dropped, gaps appeared within the network, and the remaining shorter filaments were randomly organized (Fig. 8F-I). This phenotype was consistent with partial collapse of the ARAF network when not securely connected to the apical membrane and crosslinking was weakened. We had expected that even only partially disconnecting ARAFs from the apical membrane would lead to larger, relaxed 1° cells (as observed in hybrid clonal ommatidia; Fig. 8D,E), but these cells were instead smaller, consistent with crowding by the wider neighboring LCs, which we speculate resisted 1° cells (Fig. 8F-I) as had occurred when formins were reduced across the eye (Fig. 6).

**Fig. 9.**
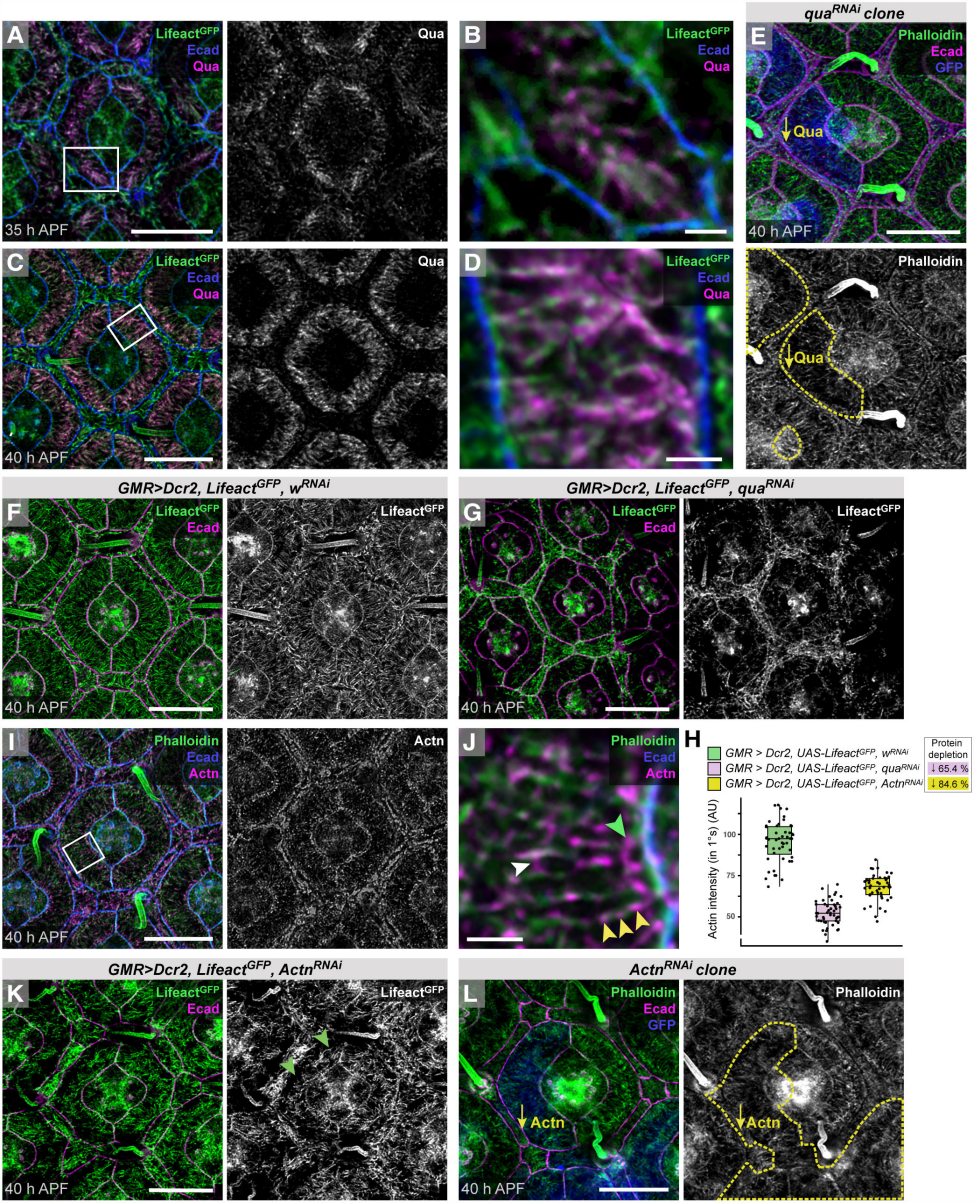
**Qua and Actn are crucial for ARAF organization.** (A,B) Immunofluorescence of Qua at 35 h APF, with boxed area shown at higher magnification in B. (C,D) Qua in an ommatidium at 40 h APF, and at higher magnification in D. (E) Hybrid ommatidium with the left 1° cell expressing *qua^RNAi^*. Phalloidin labels F-actin. Yellow dashed line outlines tissue with RNAi transgene expression, marked by GFP (in blue). (F,G) Control retina with *w^RNAi^* (F) and with *qua^RNAi^* (G) expression. F is also shown in Fig. S11. (H) Analyses of apical-cortical F-actin density; box plot indicates the median and quartiles. See Table S11 for statistical analyses and sample numbers. (I,J) Actn detected at 40 h APF, with boxed area enlarged in J. Yellow arrowheads highlight periodic distribution of Actn along an ARAF; white arrowhead indicates Actn linking ARAFs; green arrowhead highlights Actn accumulation toward AJs. (K) Small region of retina with widespread *Actn^RNAi^* expression; green arrowheads indicate gaps between ARAF network and the ZA. (L) Hybrid ommatidium with the left 1° cell expressing *Actn^RNAi^*, labeled as for the ommatidium in E. Scale bars: 1 µm (B,D,J); 10 µm (A,C,E-I,K,L). AU, arbitrary units.

Villin proteins are primarily actin bundlers, but villins have also been shown to tether actin to the membrane and sever or nucleate actin, depending on their post-translational modification (Khurana and George, 2008; Sharkova et al., 2023). We found that the *Drosophila* villin Quail (Qua) (Mahajan-Miklos and Cooley, 1994) was expressed at high levels in 1° cells and accumulated along ARAFs as they formed (Fig. 9A,B). By 40 h APF, virtually no Qua was observed in LCs, and Qua was highly enriched along ARAFs (Fig. 9C,D). Expression of *qua^RNAi^* transgenes rendered the ARAF network severely eroded but invariably led to smaller 1° cells (Fig. 9E-H), which appeared to be because cell viability was compromised: fewer than nine LCs surrounded many ommatidia, which often lacked one 1° cell (Fig. S10), indicating that cells had died in response to *qua^RNAi^* expression. Smaller 1° cells in *qua^RNAi^* hybrid ommatidia or *GMR>qua^RNAi^* retinas may accordingly have begun to die. Nonetheless, the random organization and sparsity of the remnant ARAF network in these 1° cells is consistent with collapse of the ARAF network consequent to loss of bundling by Qua, and compromised tethering to the apical membrane. Hence, we posit that ARAFs are not individual actin filaments but rather bundles, and we therefore refer to them as fibers.

Our search for other actin-bundling proteins expressed in 1° cells also identified α-Actinin (Actn) (Fyrberg et al., 1990), which localized to ARAFs (Fig. 9I). Close inspection revealed periodic distribution of Actn along ARAFs (Fig. 9J, yellow arrowheads), bridging gaps between neighboring ARAFs (Fig. 9J, white arrowheads), and accumulating on ARAFs towards ZAs (Fig. 9J, green arrowheads). Our observations are consistent with Actn localization to F-actin, stress fibers, and ZAs in other fly and vertebrate cells (Lopez-Gay et al., 2020; Pradhan et al., 2023; Rodriguez-Diaz et al., 2008; Wahlstrom et al., 2004) (reviewed by Rajan et al., 2023; Sjoblom et al., 2008). Reducing Actn across the eye (by 84.6%) reduced the density of apical cortical actin and the remaining fibers were shorter, curled, or randomly oriented, consistent with a role for Actn in crosslinking ARAFs to organize and maintain the network (Fig. 9H,K). Simultaneously reducing Qua and Actn devastated the ARAF network (Fig. S11). Reducing Actn generated many small gaps between the remaining ARAF network and the 1° cell boundary (Fig. 9K, green arrowheads), similar to the gaps observed when we reduced Ecad or α-Cat, although less severe. This suggests that Actn may contribute to linking the ARAF network to ZA-actin or AJs. Consistent with this role, reducing Actn in one 1° cell of an ommatidium enlarged that 1° cell (Fig. 9L, Fig. S7). These data underscore that crosslinking of actin bundles into an ARAF network, securing the network to the apical membrane, and tethering the network to ZAs, is essential for integrity of the network and to correctly size 1° cells.

### ARAFs introduce or support curvature of the apical membrane of 1° cells

**Fig. 10.**
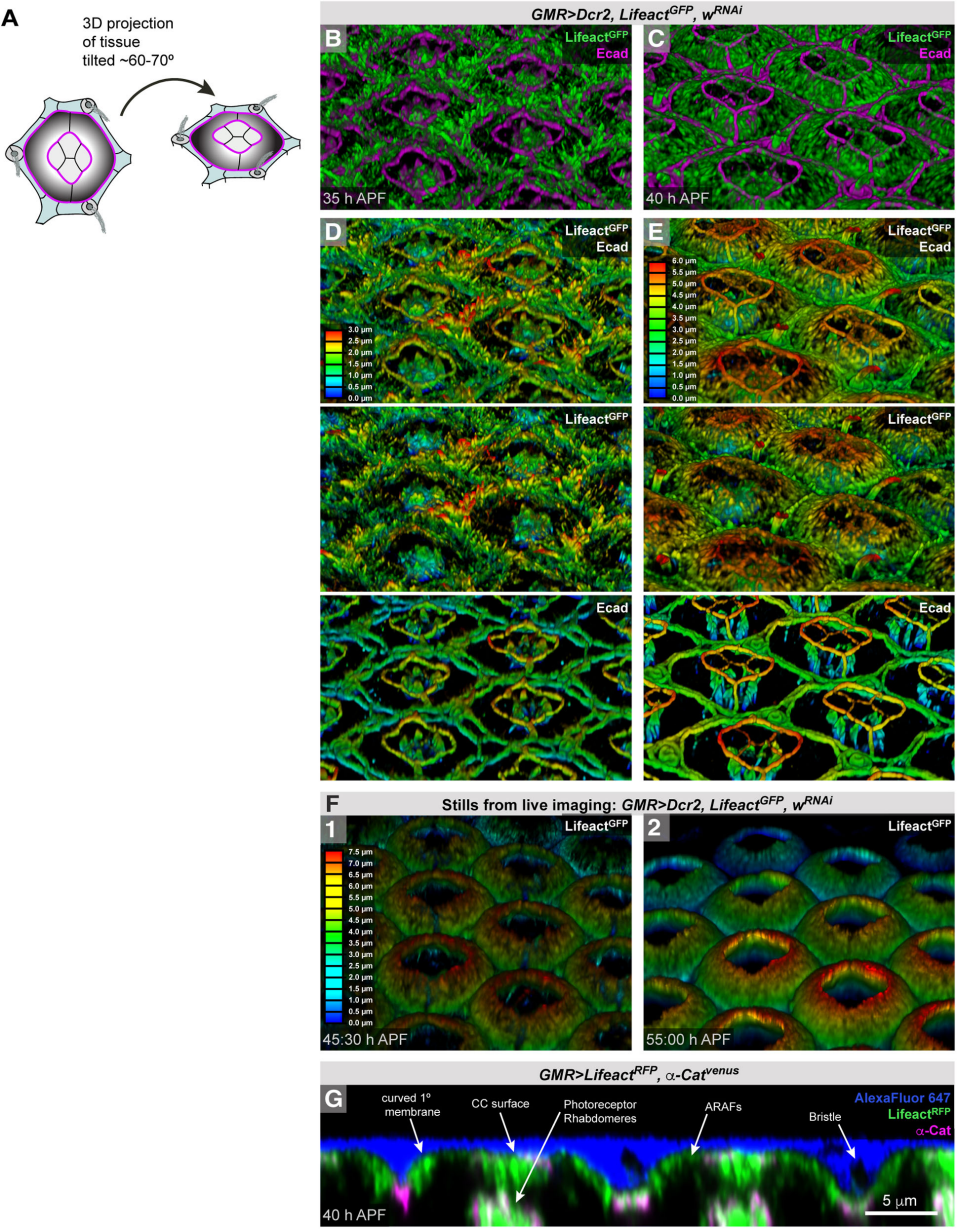
**Ommatidia become domed as they mature.** (A) Diagram of an ommatidia from above (left) and tilted at roughly the same angle as 3D perspective images in B-F (right). (B-E) 3D perspective images of the eye at 35 h APF (B) and 40 h APF (C), with Lifeact^GFP^ and Ecad. In D and E, the images from B and C are presented with depth color-coding for Lifeact^GFP^ plus Ecad; Lifeact^GFP^ only; Ecad only. (F) 3D depth-coded images of F-actin in an eye imaged live at 45:30 h APF and 55 h APF (from Movie 2). (G) Longitudinal cross-section through the apical region of an eye, with Lifeact^RFP^ and α-Cat detected and the extracellular media labeled with Alexa Fluor^®^ 647. Scale bar: 5 µm.

Given the three-dimensional nature of the cytoskeleton, we next considered the structure of the ARAF network from this perspective. 3D renderings of our confocal images revealed that in the *Drosophila* retina the apical cytoskeleton, and by inference the membrane to which it is tethered, changes dramatically from 35 to 40 h APF (Fig. 10A-E). Specifically, by 40 h APF, when the ARAF network was well formed, 3D renderings showed that the ARAFs arched from the outer 1°:LC ZAJs to inner 1°:CC ZAJs (Fig. 10C,E). The arching morphology of fibers is referenced in the name ARAFs (R for ‘ribs’). Our renderings also revealed that numerous filopodial-like structures project apically from LCs at 35 h APF (Fig. 10B,D), but these vanished by 40 h APF (Fig. 10C,E). Hence, retraction of apical filopodia from LCs, and the formation of ARAFs, transformed the retina into a landscape of repeating ‘ommatidial domes’. After 40 h APF, ARAFs became increasingly dense and domes more pronounced (Fig. 10F, Movie 3). Unfortunately, expression of membrane tags (example, palmitoylated mKate) failed to clearly label the apical membranes of ommatidia. Instead, imaging retinas that had not been exposed to detergent, and mounted in media flooded with Alexa Fluor 647, which remained extracellular, confirmed that our 3D renderings of ARAFs reflect the domed shape of the apical surface of ommatidia (Fig. 10G). Longitudinal sections of these retinas also revealed that the apical membrane of CCs is relatively flat in comparison to the curved apical membrane of 1° cells (Fig. 10G).

**Fig. 11.**
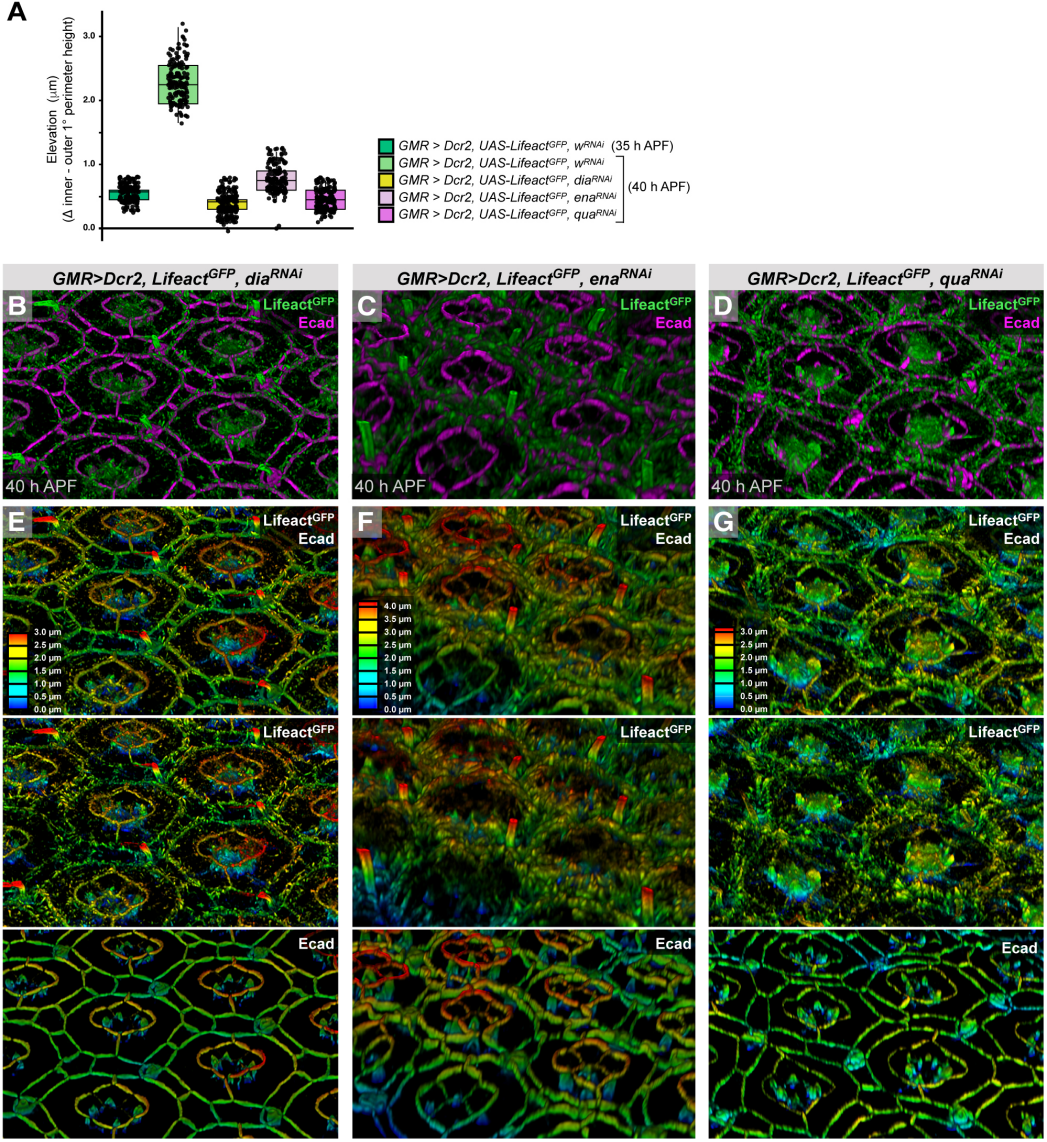
**Ommatidial doming depends on the ARAF network.** (A) Quantification of the elevation of the inner 1°:CC ZAs in relation to outer 1°:LC ZAs, at 35 and 40 h APF. Genotypes and age indicated in the key. See also Fig. S11. Box plots indicate medians and quartiles; sample numbers and statistical analyses are summarized in Table S12. (B-G) 3D perspective image of eyes expressing *dia^RNAi^* (B), *ena^RNAi^* (C) or *qua^RNAi^* (D) at 40 h APF, with Lifeact^GFP^ and Ecad detected. In E-G, the images from B-D are presented with depth color-coding for Lifeact^GFP^ plus Ecad; Lifeact^GFP^ only; Ecad only. See Fig. 10C,E for control data.

Concurrent with the formation of ARAFs, we observed that by 40 h APF the inner ZAJs of 1° cells were higher than the outer ZAJs (Fig. 10C,E). We measured this as elevation (difference between apical-basal position of inner and outer ZAJs of 1° cells), which we found increased from 0.55 µm at 35 h APF (s.d.=0.13) to 2.29 µm by 40 h APF (s.d.=0.31) (Fig. 11A, Fig. S12). To test whether ARAFs were necessary for elevation of inner junctions, we impaired the ARAF network by disrupting the formation of ARAFs (by reducing Dia; Fig. 11B,E), hampering formation of ZA-actin (by reducing Ena; Fig. 11C,F), or disabling bundling and apical anchorage of ARAFs (by reducing Qua; Fig. 11D,G). In each of these treatments, the inner ZAJs remained at the same elevation as that observed in wild-type ommatidia at 35 h APF (Fig. 11A, Fig. S12). We hence conclude that the ARAF network is built to support doming of ommatidia. During this morphogenesis, inner 1° cell junctions move apically, driven or supported by ARAFs. Earlier, we described that ARAFs constrain the transverse expansion of the fabiform shape of 1° cells. Hence, the ARAFs exert morphogenetic forces in both transverse and longitudinal axes.

### 1° cells have a complex 3D shape

**Fig. 12.**
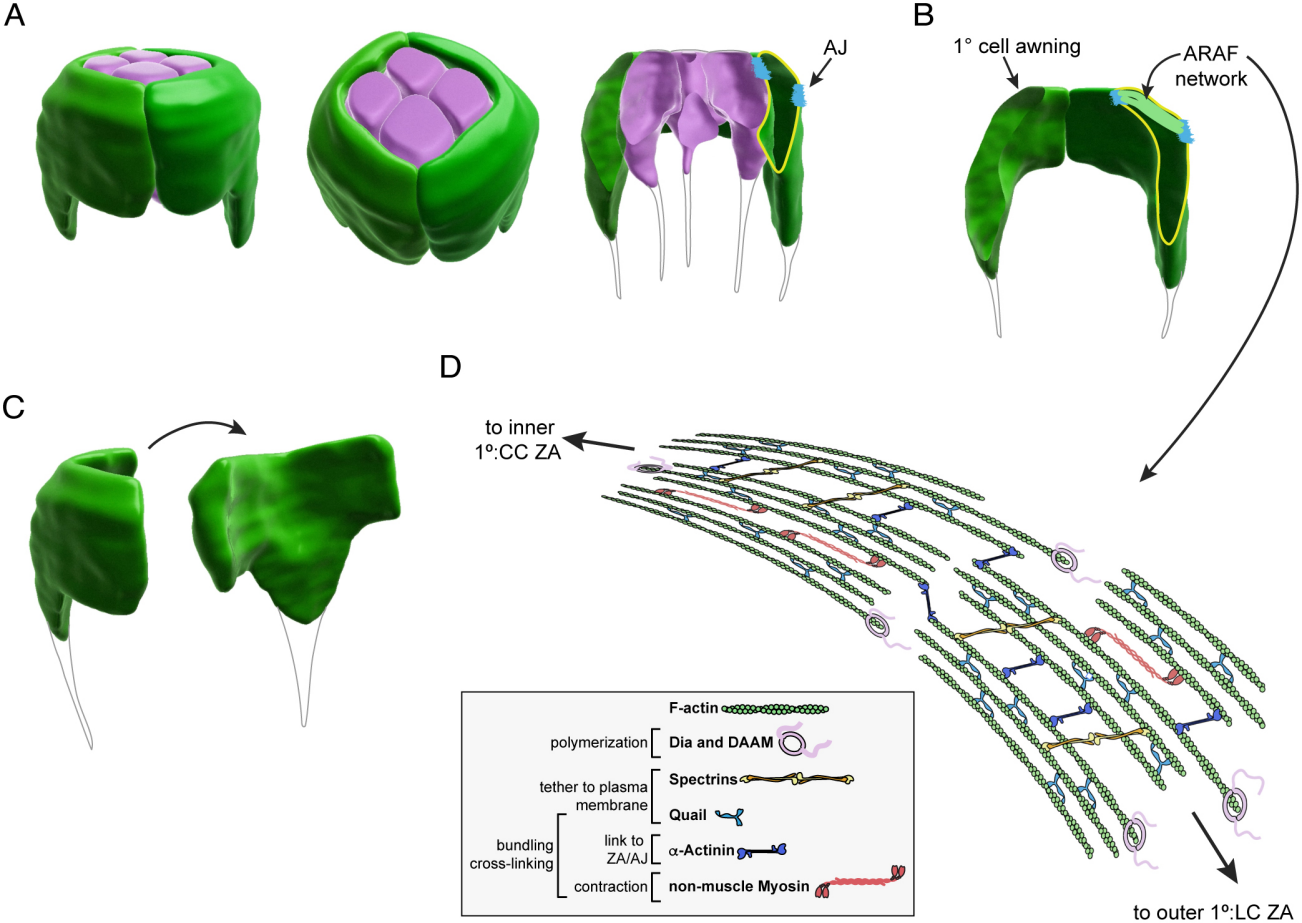
**ARAFs support the complex shape of 1° cells.** (A) 3D rendering of the cone cells (pink) and 1° cells (green) of an ommatidium. For the image on the right, the ommatidium was bisected along the midline; opening of the left 1° cell is outlined in yellow and AJs drawn in blue. (B) A pair of 1° cells, with 1° cell awning and ARAFs annotated. (C) A single 1° cell shown in two orientations. See also Movies 3-5. In A-C, basal projections of 1° cells and CCs are annotated as rendering these was less successful. (D) Model of the ARAF network with proteins responsible for its construction and maintenance.

Finally, to better understand how the ARAF network shapes 1° cells, we built 3D renderings of 1° cells from confocal *z*-stacks of α-Spec staining, which labels all membranes at 40 h APF (Fig. 12A-C, Movies 3-5). These renderings revealed that 1° cells have a complex shape not captured in traditional 2D drawings of ommatidia (Fig. 1A). The stereotypical fabiform shape is generated by widening only of the apical region of 1° cells into an ‘awning’, that reaches over and anchors to CCs. Tensile ARAFs occupy this awning, and span its width (Fig. 12B,D). Only the top third of each 1° cell connects to a partner 1° cell to form a collar around the CCs. The basal third of each 1° cell narrows and projects toward the base of the ommatidium.

## DISCUSSION

How cells acquire different cytoskeletal structures to support different cell shapes is poorly understood. We found that the apical zone of 1° cells of the *Drosophila* eye becomes filled with actin fibers organized into a complex ARAF network whilst in 3° cells and horizontal 2° cells a ‘starburst’ organization of actin emerges, and no obvious actin organization becomes discernible in oblique 2° cells. Each of these cells have distinct shapes and occupy particular positions, and we predict that the cell cytoskeletal architecture is in large part the mechanical response to position-dependent forces transmitted to that cell. However, our interrogation of 1° cells identified a comprehensive set of proteins that assemble and maintain the ARAF network (Fig. 12D) and several of these (Kst, Qua) were specifically enriched in 1° cells, underscoring that the mechanical response of these cells is pre-determined by genetic control. How Kst and Qua are regulated to become enriched in 1° cells was not explored.

Higher resolution imaging is required to confirm the precise structure of the ARAF network. However, our data support a model in which ARAFs are composed of actin bundles that connect to span the width of 1° cells and connect laterally to generate an organized network (Fig. 12D). 3D renderings of the network (Fig. 10) showed that ARAFs are curved and that the network arches upward from outer ZAJs (between 1° cells and LCs) toward the inner ZAJs (between 1° cells and CCs). ARAFs occupy only the apical region of 1° cells, which is enlarged and bends to generate an ‘awning’ that partially covers the neighboring CCs. Compromising the formation or maintenance of ARAFs, or their connection to 1° cell ZAs, led to flattened 1° cells with awnings that failed to acquire their consistent width and rounded shape (Fig. 11). Hence the ARAF network is essential for 1° cells and ommatidia to be correctly shaped.

The formins Dia and DAAM function to assemble fibers of the ARAF network. This is supported by the presence of these formins at the emergent tips of ARAFs and severe loss of ARAFs when Dia and/or DAAM activity were compromised (Figs 5 and 6). It is not clear how nucleation of ARAFs is initiated, but it is plausible that elements of the apical-medial actin meshwork present in 1° cells prior to ∼33 h APF are reorganized to form the first ARAFs, or instead severed to generate short fragments then elongated into ARAFs. Alternatively, ARAFs could be generated entirely *de novo* by membrane-anchored formins, as actin cable are generated in nurse cells (Logan et al., 2022). Indeed, Dia, and later DAAM, were enriched at AJs so these could be key sites from which ARAFs form (Fig. 5).

ARAFs are linked to the apical membrane and crosslinked into an organized network that spans the 1° cell width. This statement is supported by our detection of spectrins and the villin Qua along nascent and maturing ARAFs and detection of Actn along and between ARAFs (Figs 8 and 9). Spectrins function primarily to tether actin to plasma membranes, but they also link actin filaments to each other (Leterrier and Pullarkat, 2022; Lorenzo et al., 2023). Villins are primarily actin bundlers, but they can also link F-actin to membranes (Khurana and George, 2008; Sharkova et al., 2023). Actns are actin crosslinkers that also link F-actin to junctions (Rajan et al., 2023; Sjoblom et al., 2008). Our model posits that these proteins function in these ways to assemble or maintain the ARAF network, but more detailed analyses are needed to test these assumptions. Nonetheless, reducing expression of each of these proteins generated phenotypes consistent with failure to bundle F-actin into ARAFs (*qua^RNAi^* expression), failure to link neighboring or tandem ARAFs to each other (*Actn^RNAi^*), failure to link ARAFs to AJs (*Actn^RNAi^*), and disconnection of ARAFs from the apical membrane (*qua^RNAi^*, *kst^RNAi^* or *α-Spec^RNAi^*) (Figs 8 and 9). The severity of each phenotype we observed would have been dependent on the degree to which target proteins were reduced and the extent to which the ARAF network was maintained by non-targeted proteins.

Renderings of the 3D shape of the ARAF network revealed that the network is curved and arches from the outer AJs between 1° cells and LCs, upward toward inner 1°:CCs AJs (Fig. 10). Renderings of the 3D shape of 1° cells confirmed that the apical membrane of 1° cells curve upward to generate domed ommatidia (Fig. 12). Hence, the ARAFs are like rafters of a roof: periodically spaced, supporting and shaping the membrane above. Curiously, disrupting ARAFs led to a change in the placement of the inner AJs: these were considerably lower and the ommatidia consequently lower. Hence the ARAF network regulates the position of the inner 1°:CCs AJs.

The ARAF network is linked to the ZAs of 1° cells. This statement stems from observations that ARAFs appear to synapse with ZA-actin or AJs (Figs 2 and 3), and multiple genetic manipulations support this interpretation. First, disrupting AJs by reducing Ecad or α-Cat ‘unhooked’ the ARAF network from the 1° cell membrane, except where AJs remained (Fig. 7). Second, reducing Actn introduced gaps between the ARAF network and the ZAs (Fig. 9), consistent with Actn linking actin to AJs, as established in other systems (Knudsen et al., 1995; Nieset et al., 1997). Third, reducing Ena, which is required for actin assembly at the ZAs (Baum and Perrimon, 2001; Leerberg et al., 2014; Scott et al., 2006), disrupted the ARAF network (Fig. 6), underscoring that ARAFs must be hooked to stable the ZAs to maintain ARAF network organization.

Decoupling the ARAF network from AJs provided strong evidence that ARAFs are tensile stress fibers. Specifically, reducing Ecad or α-Cat disconnected ARAFs from the outer 1°:LC AJs and the network retracted into a rounded shape mimicking the fabiform shape of 1° cell awnings, whilst the disconnected outer membranes of 1° cells billowed outward (Fig. 7). These experiments are a genetic equivalent to laser ablation and convey that a crucial role for the tensile ARAF network is to limit the girth of 1° cells and shape each 1° cell pair into an almost-perfect ring. Hence, returning to our analogy of ARAFs as rafters, the ARAFs are also like rafter ties that prevent the walls of a house from splaying outward.

Apical stress fibers have been described in two other *Drosophila* tissues: the thoracic and tracheal epithelia (Hannezo et al., 2015). In the thorax, stress fibers form in response to pulling mechanical forces (Lopez-Gay et al., 2020). Mechanical forces could similarly induce or modify ARAF formation and density. Indeed, we found that ARAF emergence correlated with the transformation of 1° cells from scalloped to rounded fabiform cells, and this transformation could provide a pulling force that induces organization of the cytoskeleton into the ARAF network. Further, changes in the ommatidial core (example, elongation of photoreceptors) or narrowing of the LC lattice as it matures could provide additional mechanical forces to 1° cells that cause the ARAF network to be fortified. Recent modeling suggested that in tracheal epithelial cells the stress fiber network is self-assembled from small ‘nanoclusters’ of F-actin in response to anisotropic cortical stress (Sekine et al., 2024). In this model, the availability of actin nanoclusters and crosslinking proteins and the rate of actin turnover governed the morphology of the stress fiber network built. Similar rules could govern how the ARAF network is shaped, with the availability and activity of Rho, Dia, DAAM, Ena, Actn, spectrins and Qua determining ARAF architecture. Our illustration of the network in Fig. 10D attempts to convey some of the geometry of the network, but fine-scale analyses are needed to determine its true organization.

This study underscores the importance of properly understanding the 3D morphology of the cytoskeleton and cells when interrogating the development of these cells. Our 3D rendering of 1° cells shows that these are not simple fabiform cells that pair to form neat rings around CCs. Instead, 1° cells are shaped like shark teeth: the top third of each 1° cell is expanded and the bottom two-thirds narrows to project to the base of the ommatidium. ARAFs fill the apical third of 1° cells to support and maintain the broad fabiform shape and support elevation of the apical plasma membrane to generate domed ommatidia. We predict that similarly extraordinary actin conformations will become evident in other tissues as the 3D morphologies of their cells are explored.

## MATERIALS AND METHODS

### *Drosophila* stocks and genetics

All crosses and *Drosophila* lines were maintained at 25°C. *Drosophila* lines used are listed in Tables S13 and S14. F-actin was detected in retinas of *w^1118^* or *ubi-Lifeact^YFP^* (Santa-Cruz Mateos et al., 2020), or retinas with *UAS-Lifeact^GFP^*, *UAS-Lifeact^RFP^* or *UAS-GMA* (Bloor and Kiehart, 2001; Hatan et al., 2011; Huelsmann et al., 2013) driven by *GMR-GAL4*, which initiates expression in eye cells after passage of the morphogenetic furrow (Freeman, 1996). *GMR-GAL4, UAS-Lifeact^GFP^*, *GMR-GAL4, UAS-Lifeact^RFP^* or *UAS-Dcr2; GMR-GAL4, UAS-Lifeact^GFP^* (abbreviated to *GMR>Lifeact^GFP^*, *GMR>Lifeact^RFP^* and *UAS-Dcr2; GMR> Lifeact^GFP^*, respectively) were crossed to UAS transgenics to reduce proteins of interest (see below). The coinFLP system was used to generate clones of eye cells with *UAS-RNAi* transgene expression (Bosch et al., 2015).

### RNA interference

To target genes of interest, all *UAS-RNAi* transgenes available from the Bloomington *Drosophila* Stock Center and Vienna *Drosophila* Resource Center at commencement of this project were obtained (Table S14) with the exception of RNAi transgenes for *Ecad* and *α-Cat* (Seppa et al., 2008). Transgenics were crossed to *GMR>Lifeact^GFP^* and *UAS-Dcr2; GMR> Lifeact^GFP^*, and pupal eyes of progeny assayed for disruption to the ARAF network and associated eye mis-patterning. For RNAi transgenes targeting the same loci, all pupal eye phenotypes were qualitatively similar, and transgenes that most severely disrupted ARAFs were selected for the experiments presented in this work. The degree of protein knockdown by RNAi transgenes was determined by detecting the target protein with immunofluorescence in retinas with the associated transgene, and comparing to control tissue expressing a transgene against *white* (*w*) (Table S3). The actin cytoskeleton was unperturbed by *w^RNAi^* or *Dcr2* expression. Occasional errors in the placement or number of LCs were observed in retinas heterozygous for *GMR-GAL4*.

### Eye dissection and immunohistochemistry

Pre-pupae were gathered and incubated at 25°C in humidity chambers until dissection as previously described (DeAngelis and Johnson, 2019) but with the following adaptations to preserve the cytoskeleton. First, after dissection in ice-cold PBS, eye-brain complexes were fixed in 4% formaldehyde in PBS supplemented with phalloidin (1:500) on ice for 35 min. Second, tissue was maintained on ice or at 4°C whenever possible. Third, to preserve the shape of ommatidia and the ARAF network, eyes were mounted on slides surrounded by a human hair that raised the coverslip from the retinas. Fourth, retinas were imaged within a few hours of preparation.

For ‘rapid imaging’, after fixation eyes, were rinsed in ice-cold PBS and PBT (PBS with 0.15% Triton X-100), incubated in mounting medium (0.5% n-propyl gallate in 80% glycerol in PBS) for 1 h, mounted on slides, and imaged immediately. Alternatively, after fixation and rinsing, eyes were dissected from optic lobes on slides, excess PBT removed, and mounting medium applied directly to the tissue, which was cover-slipped and immediately imaged.

For immunofluorescence, after fixation and rinsing, retinas were incubated with the antibodies listed in Table S15. Endogenous fluorescence was detected when imaging Actn^GFP^, DAAM^GFP^, GMA, Kst^GFP^, Lifeact^GFP^, Lifeact^RFP^, Lifeact^YFP^, shg^mTom^ and α-Spec^GFP^ (Table S13). When used for F-actin detection, rhodamine-phalloidin was included in the primary antibody solution (1:500; Thermo Fisher Scientific, R415).

Live imaging was performed as previously described (DeAngelis and Johnson, 2019), with the following modifications: pupae were positioned with their abdomens and head on a support of soft paper putty, and surrounded by a circle of putty onto which the coverslip was placed. The coverslip was depressed to reach but not touch the eye.

### Protein localization

Localization of ARAF-associated proteins was assayed with primary antibodies (Table S15) and confirmed by GFP-fusion transgenics or reporter transgenes if available (Table S13). Immunofluorescence was compared in retinas with and without RNAi transgenes targeting the detected protein (Table S14; Fig. S6).

### Image acquisition and processing

High-resolution *z*-stack images were acquired with a Leica SP8 confocal microscope and proprietary Leica Application Suite X software (LASX) and LIGHTNING deconvolution. Images were gathered with a scan format of 2048×2048 and resolution of 45 nm×45 nm per pixel. Control and experimental retinas were imaged using precisely the same parameters. Maximum projections of sections of the apical region of 1° cells were generated in LASX and processed in Adobe Photoshop^®^, with control and experimental images processed identically. To visualize ARAF architecture, *z*-stacks of Lifeact^GFP^ were rendered as 3D and depth-coded images using the 3D feature of LASX. To determine the 3D architecture of 1° cells, *z*-stacks of α-Spec immunofluorescence were rendered as 3D images using the surface feature of Imaris (Oxford Instruments).

### Image analysis and statistics

To determine the orientation of ARAFs with respect to the 1°:LC boundary (Tables S1 and S10), the angle tool of Fiji (Schindelin et al., 2012) was used to trace individual filaments and the adjacent outer 1° cell ZA, and angles then measured. Neighboring filaments were similarly traced and the angle tool used to measure the angle of orientation between filaments within the ARAF network.

To determine ARAF density (Table S2), six 2 µm lines were drawn per 1° cell, parallel to the outer perimeter of 1° cells. Fiji's plot profile function was then used to generate plots of fluorescence intensity of Lifeact^GFP^, Lifeact^RFP^, Lifeact^YFP^ or rhodamine-phalloidin along each line, with each plot peak representing an intersected actin filament. The number of ARAFs/µm was then calculated.

To determine how severely RNAi transgenes reduced target proteins (Table S3, Fig. S6), the apical outlines of 1° cells were traced with the polygon tool of Fiji and mean fluorescence intensity of the protein, detected with immunofluorescence, was determined in images of retinas with and without transgene expression.

For phenotypic analyses of 1° cells after RNAi transgene expression (Tables S4-S8, S11-S13), the polygon tool was used to trace the apical outlines of 1° cells area then measured. For control retinas, measurements were normalized to generate mean area values of 100 arbitrary units. For experimental data, each 1° cell area measurement was normalized against the mean area of control 1° cells. Actin density was then determined in the entire 1° cell as mean intensity of Lifeact^GFP^. To measure actin density at ZAs, Fiji's polygon tool was used to trace the outline of 1°:LC AJs that had been detected with Ecad immunofluorescence and intensity of Lifeact^GFP^ within these outlines was then determined. For controls, mean intensity measurements were normalized to generate a mean of 1 AU and experimental measurements were normalized against the mean actin intensities of controls. In 1° cells in which ARAFs had been released from the ZA, the polygon tool was used to outline the remaining ARAF network, and the area then measured (Table S8).

Elevation of ommatidial domes was calculated as the difference in the position of the confocal *z*-section that captured the top of AJs between 1° and CCs, and the position of the *z*-section that captured the 1° to LC AJs. Pooled elevation data are presented in Fig. 9A, detailed analyses in Fig. S6.

Statistically significant differences between genotypes were determined using unpaired two-tailed Student's *t*-tests; *P*-values and sample sizes are listed in Tables S1-S12. All graphs were generated using R software.

## Supplementary Material

10.1242/develop.204931_sup1 Supplementary information
