# Supplementary material for: Generation and maintenance of apical rib-like actin fibers in epithelial support cells of the Drosophila eye
Source: Development. 2026 Jan 8;153(1):dev204931. doi: 10.1242/dev.204931 (PMC12848579; doi:10.1242/dev.204931)
Supplement: Supplementary information [file develop-153-204931-s1.pdf]

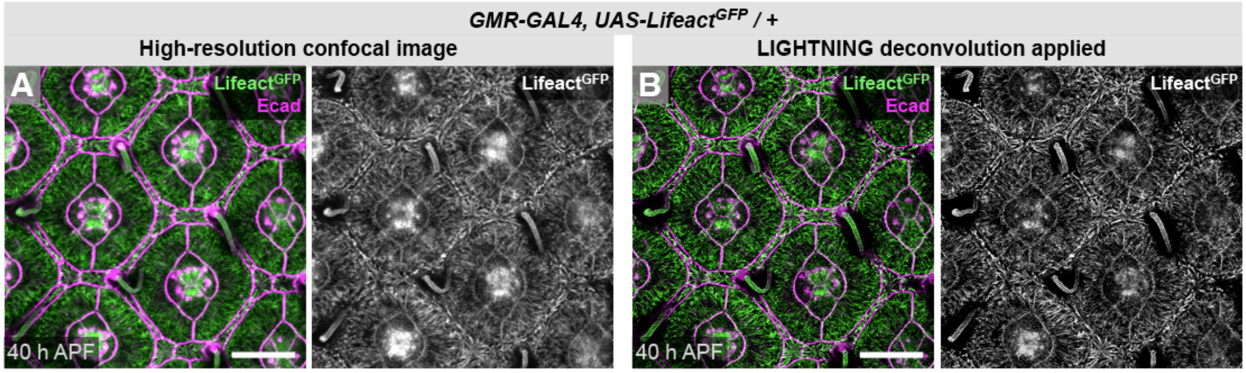

Fig. S1. SP8 LIGHTNING deconvolution enabled detailed observation of the cytoskeleton in the pupal eye. (A) Confocal image of a small region of an eye dissected at 40 h APF and (B) the same image after application of LIGHTNING deconvolution to remove signal noise and better resolve actin filaments. Scale bars: 10 μm.

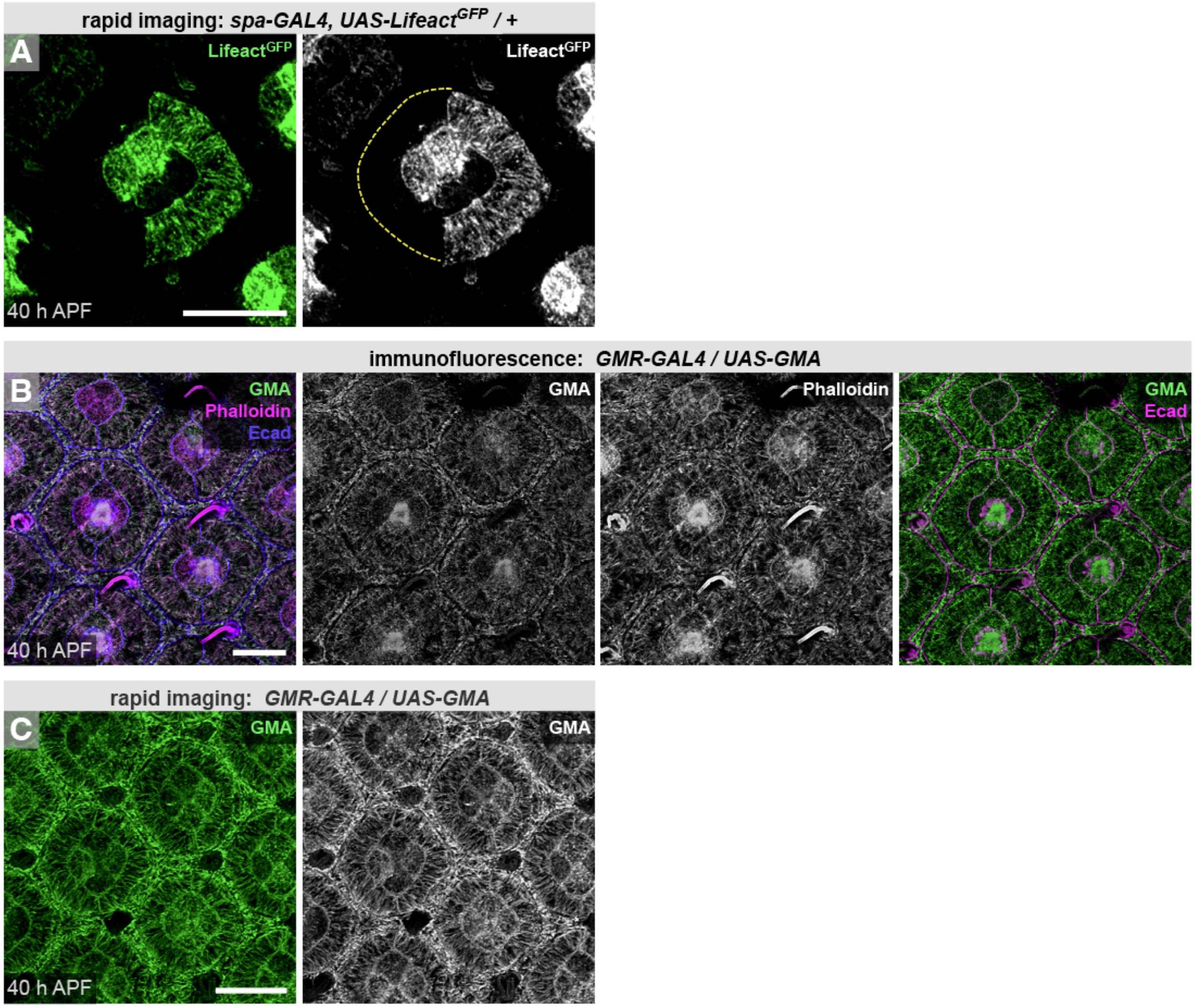

Fig. S2. Detection of F-actin at 40 h APF. (A) Mild expression of *Lifeact*^{GFP} in 1° and CCs with *spa-GAL4*. Expression of *GMA* with *GMR-GAL4* and (B) imaged after detection of Ecad and incubation with phalloidin, or (C) rapid imaging. GMA fluorescence survived immunofluorescence protocols poorly.

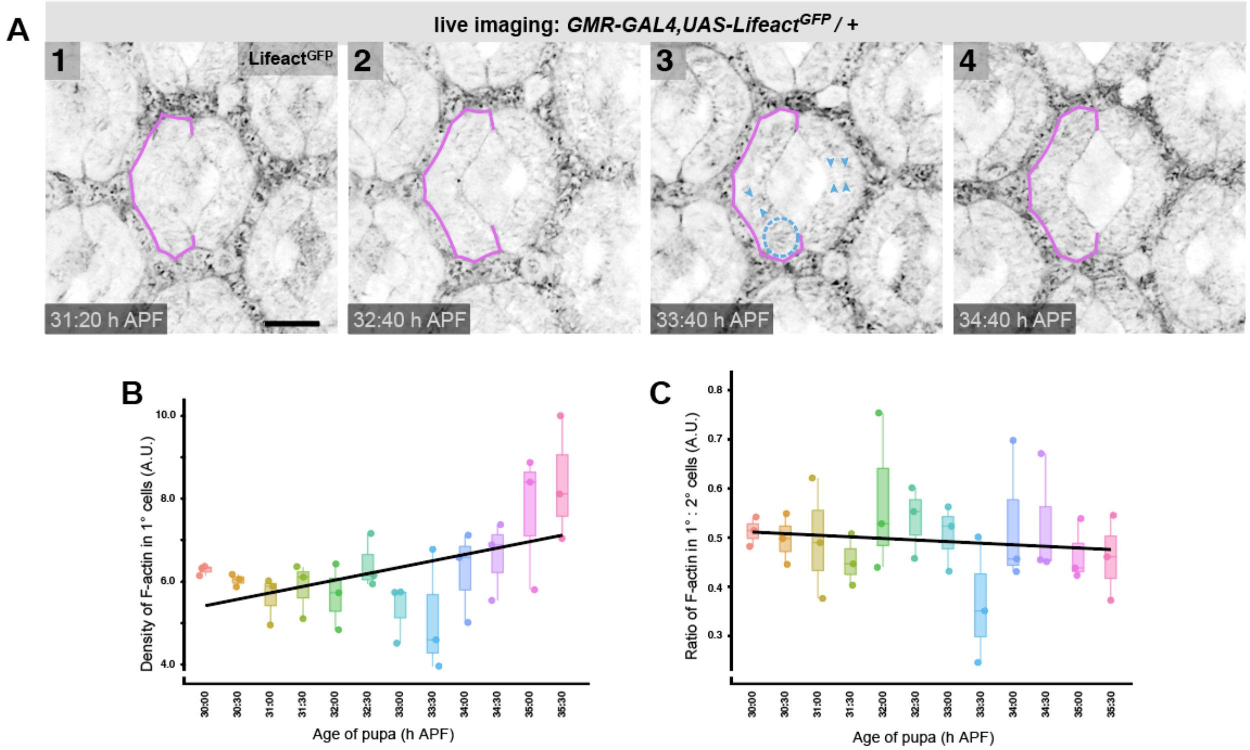

Fig. S3. Filaments of the ARAF network appear from about 33 h APF. (A) Stills from a retina imaged live, with age indicated. Panel 2 and 3 are also presented in Fig. 3C. One 1° is outlined in magenta to highlight shape of the 1° cell. Blue arrowheads indicate groups of short ARAFs oriented in parallel. Dotted circle outlines an whisker-like arrangement of F-actin. Analyses of (B) density of apical-cortical F-actin in 1° cells, and (C) ratio of apical-medial F-actin in 1° and 2° cells, in the live-imaged eye. Box plots indicate medians and quartiles. Scale bars: 5 μm.

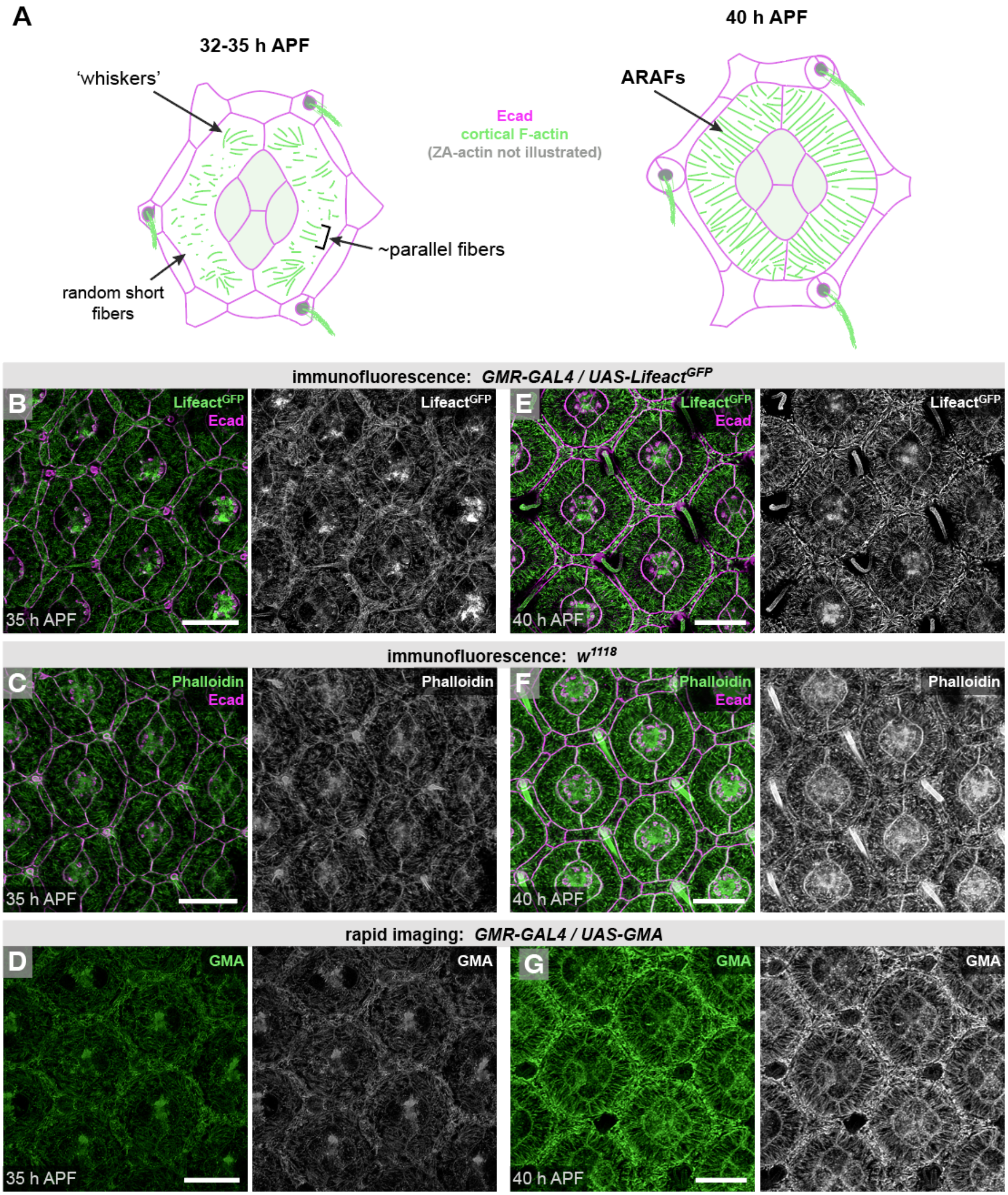

Fig. S4. F-actin at 35 h versus 40 h APF, using different detection methods. (A) Cartoons of ommatidia at 32-35 h APF and 40 h APF, summarizing apical-cortical F-actin in 1°s. F-actin detected via (B) *Lifeact^{GFP}*, (C) phalloidin, and (D) *GMA* at 35 h APF and 40 h APF (E-G). Note (E) is also presented as Fig 2A, (F) also presented as Fig. 2F and (G) as Fig. S2C, but repeated here to facilitate comparison with detection at 35 h APF. Scale bars: 10 µm.

Development • Supplementary information

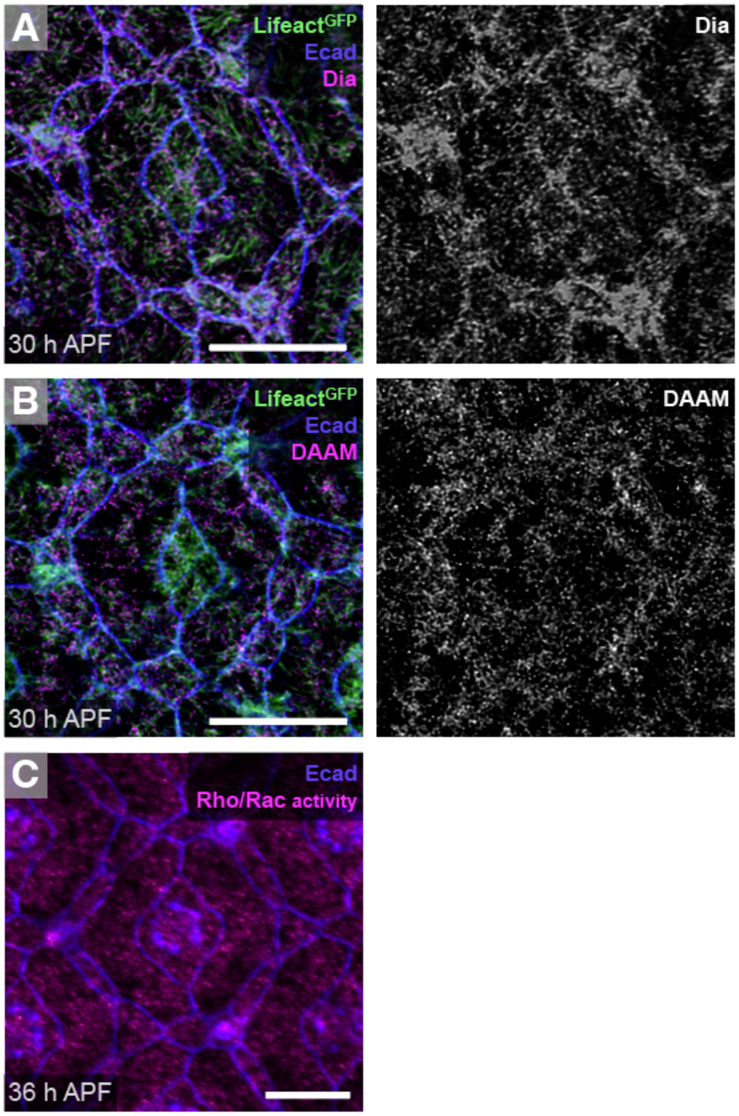

Fig. S5. Formins are expressed in 1° cells prior to ARAF emergence.

Immunofluorescence of (A) Dia and (B) DAAM at 30 h APF, before ARAFs emerge. Ecad labels AJs and $Lifeact^{GFP}$ labels the cytoskeleton. (C) The Rho activity sensor $Pkn.RGD^{G58A\text{-}eGFP}$ (shown in magenta) localized through the apical region of 1° cells. Scale bars: 10 μm.

Development • Supplementary information

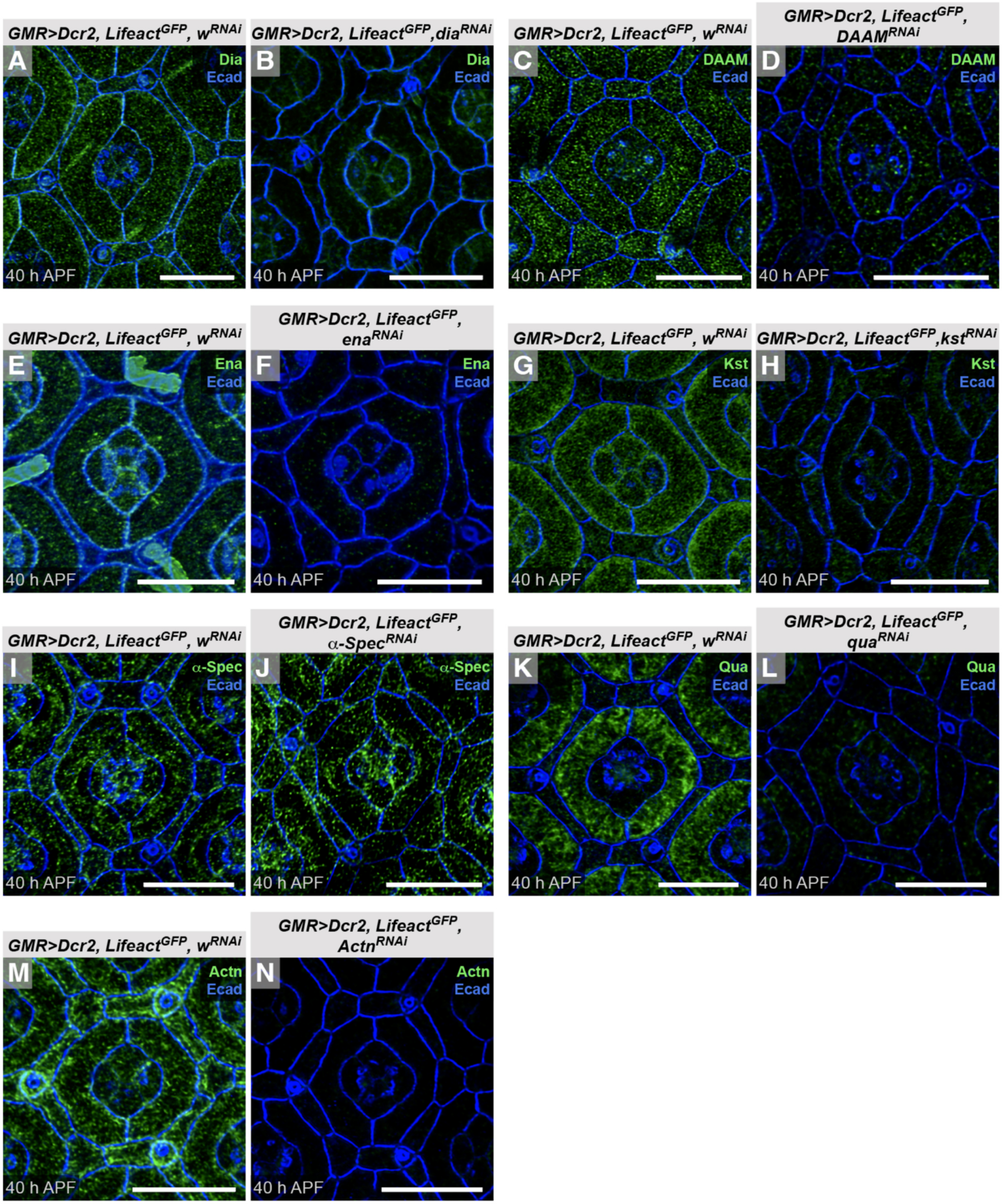

Fig. S6. Immunofluorescence of protein components of the ARAF network when targeted by RNAi transgenes. Ommatidia in control ommatidia (with w^{RNAi} expression) and experimental ommatidia with RNAi transgenes as shown. Ecad and target proteins detected in each panel, as indicated. Scale bars: 10 μm.

Development • Supplementary information

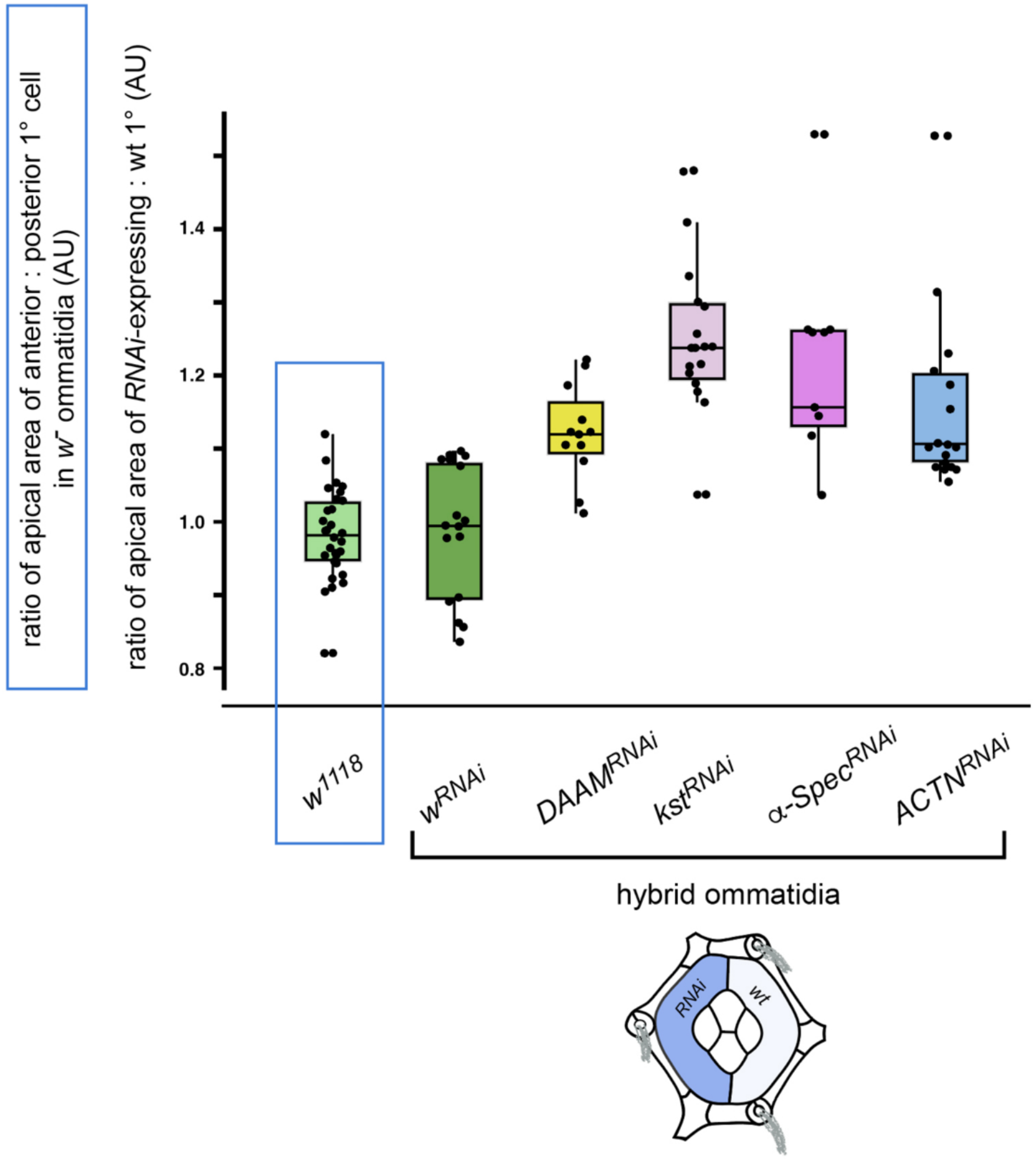

Fig. S7. Comparison of the area of partner 1° cells in an ommatidium. Box and whisker plots (indicating medians and quartiles) of the ratio of anterior to posterior 1° cell apical area, and *RNAi* to wild type 1° cells in hybrid ommatidia generated as clones (as illustrated below plot).

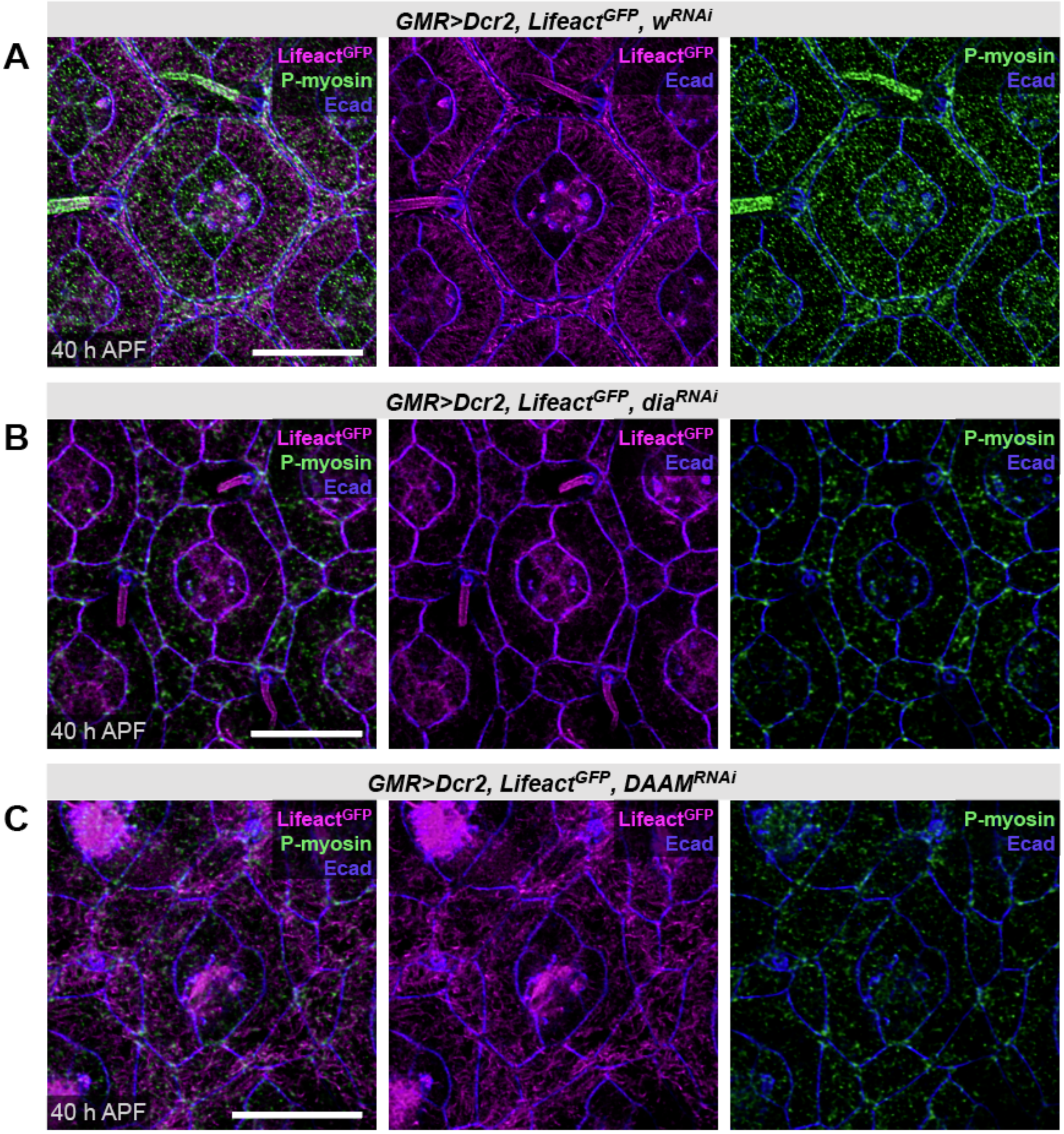

Fig. S8. Disrupting ARAFs reduced NMII activity. (A) Ommatidium with w^{RNAi}, (B) dia^{RNAi} or (C) $DAAM^{RNAi}$. $Lifeact^{GFP}$ (in magenta) and phosphorylated myosin report F-actin and activated NMII respectively. Scale bars: 10 μm.

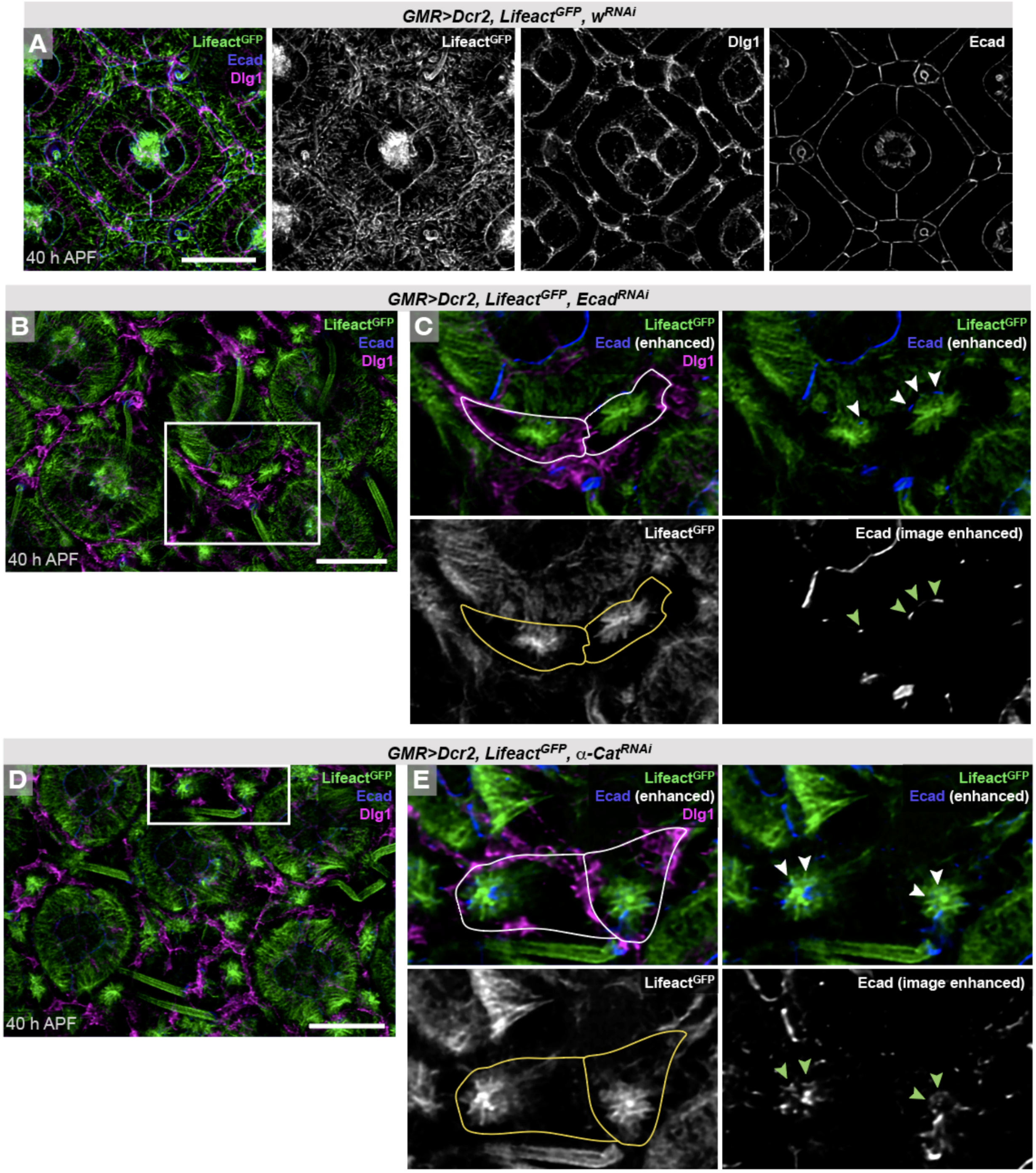

Fig. S9. Integrity of AJs is essential for formation of the apical cytoskeleton in lattice cells. (A) Control retina with *w*^{RNAi} expression. (B) Retina with *Ecad*^{RNAi}, image also presented in Fig. 7A. Lifeact^{GFP} labels F-actin, Ecad marks AJs and Dlg labels lateral membranes. Boxed region shown at higher magnification in (C). (D) Retina with α-*cat*^{RNAi}, with boxed region at higher magnification in (E). Image also presented in Fig. 7B. In (C) and (D), two LCs are outlined and arrowheads indicate extant AJs or internalized Ecad associated with F-actin tufts. Scale bars: 10 µm.

Development • Supplementary information

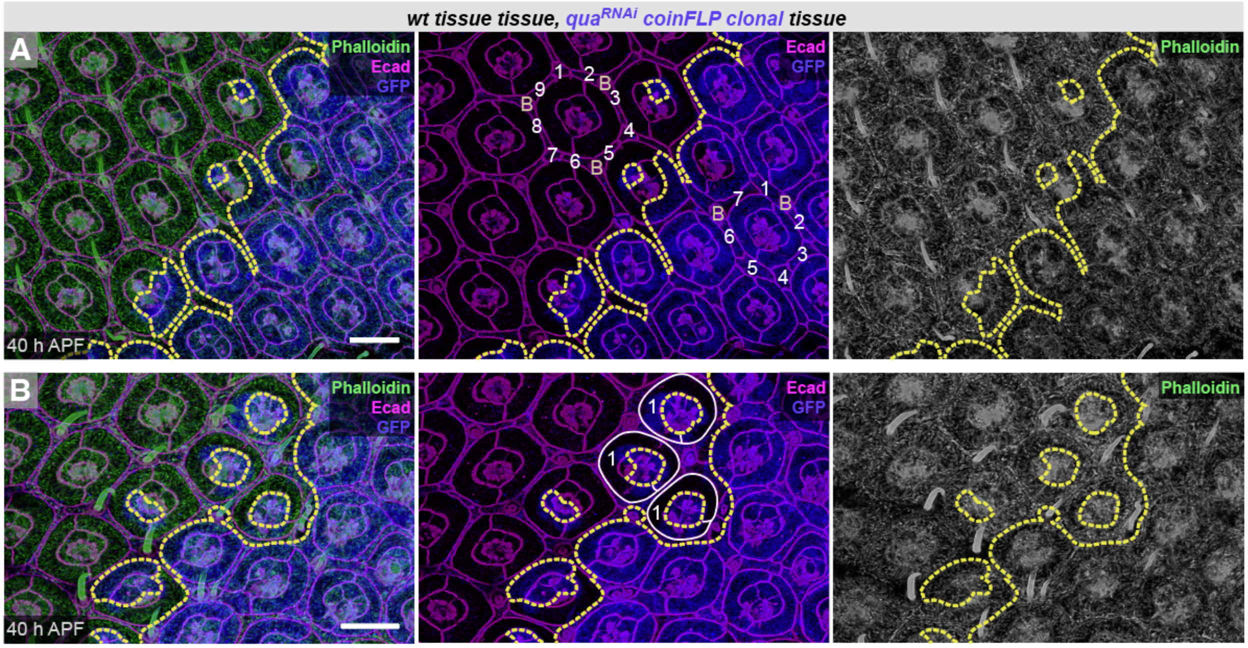

Fig. S10. Reducing Qua compromises cell viability. (A) and (B) Two examples of clones of eye tissue with *qua*^{RNAi} expression, marked by GFP (in blue). Wild-type ommatidia are typically surrounded by nine LCs and three bristle groups (indicated in central image, panel A). Fewer LCs surrounded all ommatidia in *qua*^{RNAi} tissue, and multiple ommatidia had one rather than two 1°s (central image, panel B), indicating that many *qua*^{RNAi} expressing cells had died. Scale bars: 10μm.

Development • Supplementary information

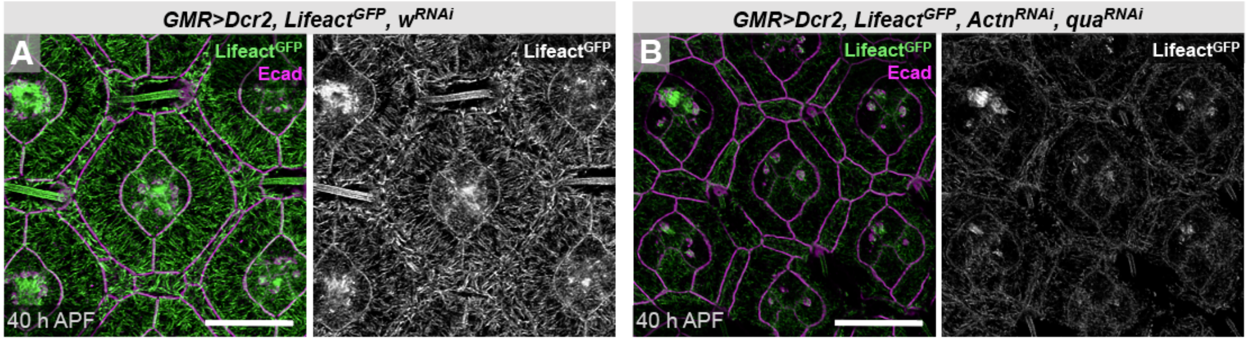

Fig. S11. Reducing Qua and Actn obliterated the ARAF network. (A) Control ommatidium with *w*^{RNAi} expression, and (B) an ommatidium with *qua*^{RNAi} and *Actn*^{RNAi} expression. Image in (A) is also presented in Fig. 9F. Scale bars: 10μm.

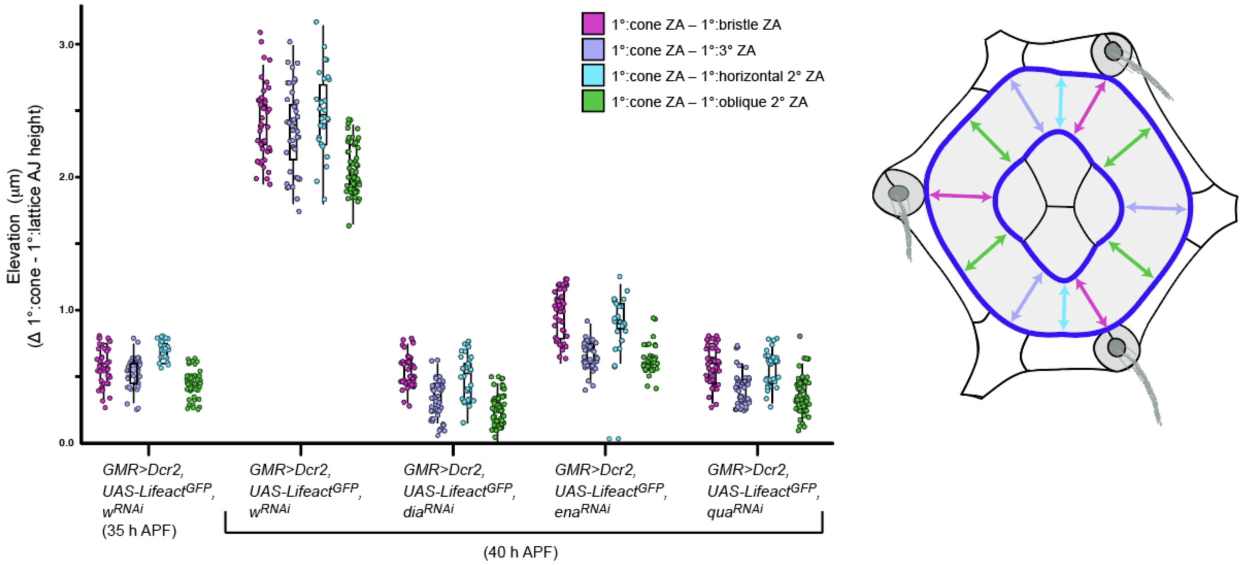

Fig. S12. Analyses of the elevation of the inner 1°:CC ZA, relative to the outer 1°:LC junctions. Separate measurements of the change in elevation of the 1°:CC ZA, with respect to four lattice cell types, as per key and illustration at right. These data reveal that the elevation from oblique 2° cells to the 1°:CC ZA tends to be lowest and least variable.

Table S1. Students' T-test p-values comparing angles of orientation of ARAFs (relates to Fig. 2H).

	ubi-Lifeact^{YFP} (n=260)	w^{1118}* (n=254)	*GMR>GMA* (n=266)	*GMR>Lifeact*^{RFP} (n=260)
GMR>Lifeact^{GFP} *(n=255)*	0.605	0.92	0.682	0.096
ubi-Lifeact^{YFP}		0.518	0.479	0.028
w^{1118}*			0.688	0.092
GMR>GMA				0.264

* ARAFs detected with rhodamine phalloidin
n = number of ARAFs, analyzed in 20 to 26 1° cells per genotype.

Table S2. Students' T-test p-values comparing ARAF density (ARAFs/µm) (relates to Fig. 2I).

	ubi-Lifeact^{YFP} (n=125)	w^{1118}* (n=113)	*GMR>GMA* (n=196)	*GMR>Lifeact*^{RFP} (n=180)
GMR>Lifeact^{GFP} (n=200)	0.001x 10^{-12}	0.008 x 10^{-10}	0.002x10^{-18}	0.002 x 10^{-08}
ubi-Lifeact^{YFP}		0.202	0.178	0.071
w^{1118}*			0.006	0.566
GMR>GMA				0.001

* ARAFs detected with rhodamine phalloidin
n = number of 2mm sections of 1° cells analyzed, in 30 to 34 1° cells per genotype.

Table S3. Protein depletion in the apical third of 1° cells of retinas dissected at 40 h APF.

Protein targeted	Transgene expressed [1.]	# of independent eyes analyzed	# of 1° cells analyzed	Mean fluorescence (AU) [2.]	Standard deviation	Mean % protein reduction	p-value (t-test)
DAAM	w^{RNAi}	3	25	93.76	15.73	55.92	0.001 x 10^{-17}
	$DAAM^{RNAi\text{-}GD8382}$	3	25	56.56	15.25		
Dia	w^{RNAi}	3	55	95.8	18.59	83.31	0.005 x 10^{-43}
	$dia^{RNAi\text{-}KK101745}$	4	40	15.99	4.36		
Ena	w^{RNAi}	3	44	84.62	24.11	74.20	0.001 x 10^{-23}
	$ena^{RNAi\text{-}GD8910}$	3	30	21.83	2.54		
Actn	w^{RNAi}	3	44	83.45	8.32	84.60	0.003 x 10^{-54}
	$Actn^{RNAi\text{-}GD1354}$	3	43	12.85	3.5		
Qua	w^{RNAi}	3	47	48.01	4.08	65.42	0.008 x 10^{-58}
	$qua^{RNAi\text{-}KK103765}$	3	30	16.6	2.14		
Kst	w^{RNAi}	3	42	23.86	3.92	60.79	0.001 x 10^{-30}
	$kst^{RNAi\text{-}GD1472}$	3	39	9.35	1.14		
α-Spec	w^{RNAi}	3	41	56.86	5.08	29.03	0.008 x 10^{-25}
	$\alpha\text{-}Spec^{RNAi\text{-}HMC04371}$	3	37	40.31	5.46		

1. Full genotypes: UAS-Dcr2 / + ; GMR-GAL4 / UAS-RNAi transgene
2. Measurements made of standard high-resolution confocal images.

Table S4. Students' T-test p-values comparing Lifeact^{GFP} fluorescence intensity in 1° cells (relates to Fig. 6B).

	GMR>Dcr2, Lifeact^{GFP}, $dia^{RNAi\text{-}KK101745}$ (n=74)	*GMR>Dcr2, Lifeact*^{GFP}, $DAAM^{RNAi\text{-}GD8382}$ (n=42)	*GMR>Dcr2, Lifeact*^{GFP}, $ena^{RNAi\text{-}GD8910}$ (n=58)
GMR>Dcr2, Lifeact^{GFP}, w^{RNAi} (n=60)	0.005 x 10^{-48}	0.007 x 10^{-18}	0.001 x 10^{-47}

n = number of 1° cells, analyzed in 2 to 4 eyes per genotype.

Table S5. Students' T-test p-values comparing Lifeact^{GFP} fluorescence intensity at AJs of 1° cells (relates to Fig. 6C).

	GMR>Dcr2, LifeactGFP, dia$^{RNAi-KK101745}$ (n=72)	*GMR>Dcr2, Lifeact^{GFP}, DAAM$^{RNAi-GD8382}$* (n=72)	*GMR>Dcr2, Lifeact^{GFP}, ena$^{RNAi-GD8910}$* (n=62)
GMR>Dcr2, Lifeact^{GFP}, w^{RNAi} (n=71)	0.004×10^{-34}	0.003×10^{-44}	0.004×10^{-41}
GMR>Dcr2, Lifeact^{GFP}, dia$^{RNAi-KK101745}$		0.002×10^{-10}	0.005×10^{-08}
GMR>Dcr2, Lifeact^{GFP}, DAAM$^{RNAi-GD8382}$			0.155

n = number of 1° cells, analyzed in 2 to 4 eyes per genotype.

Table S6. Students' T-test p-values comparing LifeactGFP fluorescence intensity in 1° cells (relates to Figure 7C).

	GMR>Lifeact^{GFP}, α-Cat$^{RNAi-3HH}$ (n=60)	*GMR>Lifeact^{GFP}, Ecad$^{RNAi[B107A1]}$* (n=57)
GMR>Lifeact^{GFP}, w^{RNAi} (n=72)	0.006×10^{-14}	0.008×10^{-25}

Table S7. Students' T-test p-values comparing size of 1° cells (relates to Figure 7D).

	GMR>Lifeact^{GFP}, α-Cat$^{RNAi-3HH}$ (n=60)	*GMR>Lifeact^{GFP}, Ecad$^{RNAi[B107A1]}$* (n=57)
GMR>Lifeact^{GFP}, w^{RNAi} (n=51)	0.003×10^{-10}	0.003×10^{-12}

n = number of 1° cells, analyzed in 3 to 4 eyes per genotype.

Table S8. Students' T-test p-values comparing area of ARAF network (relates to Figure 7E).

	GMR>Lifeact^{GFP}, α-Cat$^{RNAi-3HH}$ (n=25)	*GMR>Lifeact^{GFP}, Ecad$^{RNAi[B107A1]}$* (n=21)
GMR>Lifeact^{GFP}, w^{RNAi} (n=36)	0.002×10^{-9}	0.005×10^{-8}

n = number of 1° cells, analyzed in 3 to 4 eyes per genotype.

Development • Supplementary information

Table S9. Students' T-test p-values comparing angles between actin filaments in 1° cells (relates to Figure 7F).

	GMR>Lifeact^{GFP}, α-Cat$^{RNAi-3HH}$ (n=131)	*GMR>Lifeact^{GFP}, Ecad$^{RNAi[B107A1]}$* (n=134)
GMR>Lifeact^{GFP}, w^{RNAi} (n=144)	0.004×10^{-14}	0.001×10^{-13}
GMR>LifeactGFP, α-Cat$^{RNAi-3HH}$		0.214

n = number of ARAFs, analyzed in 36 to 38 1° cells per genotype.

Table S10. Students' T-test p-values comparing LifeactGFP fluorescence intensity in 1° cells (relates to Fig. 8I).

	GMR>Dcr2, Lifeact^{GFP}, α-spec$^{RNAi-HMC04371}$ (n=42)	*GMR>Dcr2, Lifeact^{GFP}, kst$^{RNAi-GD1472}$* (n=70)
GMR>Dcr2, Lifeact^{GFP}, w^{RNAi} (n=96)	0.002×10^{-21}	0.001×10^{-37}

n = number of 1° cells, analyzed in 2 to 3 eyes per genotype.

Table S11. Students' T-test p-values comparing LifeactGFP fluorescence intensity in 1° cells (relates to Fig. 9H).

	GMR>Dcr2, Lifeact^{GFP}, Actn$^{RNAi-GD1354}$ (n=44)	*GMR>Dcr2, Lifeact^{GFP}, qua$^{RNAi-KK103765}$* (n=46)
GMR>Dcr2, Lifeact^{GFP}, w^{RNAi} (n=48)	0.002×10^{-24}	0.001×10^{-36}

n = number of 1° cells, analyzed in 2 to 3 eyes per genotype.

Development • Supplementary information

Table S12: Students' T-test p-values comparing elevation of 1°:cone cell AJ (in relation to 1°:lattice cell AJ) (relates to Fig. 11A).

	GMR>Dcr2, Lifeact^{GFP}, w^{RNAi} 40 h APF, n=168	*GMR>Dcr2, Lifeact^{GFP}, dia$^{RNAi\text{-}KK101745}$* 40 h APF, n=192	*GMR>Dcr2, Lifeact^{GFP}, ena$^{RNAi\text{-}GD8910}$* 40 h APF, n=153	*GMR>Dcr2, Lifeact^{GFP}, qua$^{RNAi\text{-}KK103765}$* 40 h APF, n=180
GMR>Dcr2, Lifeact^{GFP}, w^{RNAi} 35 h APF, n=180	0.002×10^{-152}	0.02×10^{-24}	0.001×10^{-27}	0.006×10^{-12}
GMR>Dcr2, Lifeact^{GFP}, w^{RNAi} 40 h APF		0.003×10^{-171}	0.001×10^{-151}	0.009×10^{-165}
GMR>Dcr2, Lifeact^{GFP}, dia$^{RNAi\text{-}KK101745}$ 40 h APF			0.006×10^{-53}	0.008×10^{-2}
GMR>Dcr2, Lifeact^{GFP}, ena$^{RNAi\text{-}GD8910}$ 40 h APF				0.001×10^{-41}

n = dome height calculated at 12 positions per ommatidium (as illustrated in Fig. S12) in 14 to 16 ommatidia per genotype.

Table S13. Drosophila lines utilized (excluding RNAi transgenics)

**Abbreviated genotype**	**Full genotype**	**Source**
α-Spec-GFP	*P{Wee-P.un}α-Spec[Wee-P]*	Gift from Claire Thomas, UPENN (Khanna et al., 2015)
Actn-GFP	*y[1] P{w[+mC]=PTT-GC}Actn[CC01961] w[*]/FM0, Bar[+]*	BDSC stock 602268
coinFLP-GAL4, UAS-GFP	*w-; P{y[+t7.7] w[+mC]=CoinFLP-GAL4}attP40 P{w[+mC]=UAS-2xEGFP}AH2*	BDSC stock 58751
ey-FLP, UAS-Dcr-2 ; Sp/CyO	*y[d2] w[*] P{ry[+t7.2]=ey-FLP.N}2 P{w[+mC]=UAS-Dcr-2.D}1; wg[Sp-1]/CyO*	BDSC stock 58756
GMR-GAL4	*w[*]; P{w[+mC]=GAL4-ninaE.GMR}12*	BDSC stock 1104
GMR>Lifeact^{GFP}	*w[*]; P{w[+mC]=GAL4-ninaE.GMR}12, P{y[+t*] w[+mC]=UAS-Lifeact-GFP}VIE-260B*	This study. Generated from BDSC 1104 and 35544.
GMR>Lifeact^{RFP}	*w[*]; P{w[+mC]=GAL4-ninaE.GMR}12, M{w[+mC]=UASp-Lifeact.TagRFP-T}ZH-22A*	This study. Generated from BDSC 1104 and 58713.
kst-GFP	*y[1] w[*]; Mi{PT-GFSTF.1}kst[MI03134-GFSTF.1]*	BDSC stock 60193
shg^{mTom}	*y[1] w[*]; TI{TI}shg[mTomato]*	BDSC stock 58789
spa-GAL4	*P{w[+mC]=spa-GAL4.J}1, w[*]*	BDSC stock 26656
sqh-Pkn.RBD$^{G58A-eGFP}$	*w[*]; P{w[+mC]=sqh-Pkn.RBD.G58A-eGFP}312a P{sqh-Pkn.RBD.G58A-eGFP}312b*	BDSC stock 52298
UAS-Dcr2	*P{w[+mC]=UAS-Dcr-2.D}1, w[1118]*	BDSC stock 24646
UAS-Dcr2; GMR>Lifeact^{GFP}	*w[*], P{w[+mC]=UAS-Dcr-2.D}1; P{w[+mC]=GAL4-ninaE.GMR}12, P{y[+t*] w[+mC]=UAS-Lifeact-GFP}VIE-260B*	This study. Generated from BDSC 1104, 35544 and 24648.
UAS-GMA	*w[1118]; P{w[+mC]=UAS-GMA}3*	BDCS stock 31776
UAS-Lifeact^{GFP}	*y[1] w[*]; P{y[+t*] w[+mC]=UAS-Lifeact-GFP}VIE-260B*	BDSC stock 35544
UAS-Lifeact^{RFP}	*w[*]; M{w[+mC]=UASp-Lifeact.TagRFP-T}ZH-22A*	BDSC stock 58713
ubi-Lifeact^{YFP}	*P{Ubi-Lifeact.YFP}*	Gift from Maria Martin-Bermudo, (Santa-Cruz Mateos et al., 2020)
w^{1118}	*w[1118]*	BDSC stock 3605

Table S14. Information on transgenic RNAi lines tested and used in this study.

Abbreviated genotype	Full genotype	Source	Previous validation	Strength of knockdown[1]	Data presented
α-Cat$^{RNAi\text{-}3F}$	*UAS-α-Cat*$^{RNAi-3F}$ */ CyO*	(Seppa et al., 2008)	(Seppa et al., 2008)	Weak	
α-Cat$^{RNAi\text{-}3HH}$	*UAS-α-Cat*$^{RNAi-3HH}$ */ TM6b*	(Seppa et al., 2008)	(Seppa et al., 2008)	Strong	yes
α-Spec$^{RNAi\text{-}GD9695}$	*w1118; P{GD9695}v25387*	VDRC stock 25387	(Deng et al., 2020)	Medium	yes
α-Spec$^{RNAi\text{-}HMC04371}$	*y[1] sc[*] v[1] sev[21]; P{y[+t7.7] v[+t1.8]=TRiP.HMC04371}attP40*	BDSC stock 56932	(Deng et al., 2020)	Strong	yes
α-Spec$^{RNAi\text{-}JF01727}$	*y[1] v[1]; P{y[+t7.7] v[+t1.8]=TRiP.JF01727}attP2*	BDSC stock 31209		Weak	
Actn$^{RNAi\text{-}GD1354}$	*w1118; P{GD1354}v7760*	VDRC stock 7760	(Clark and Kadrmas, 2013)	Strong	yes
Actn$^{RNAi\text{-}GD1354}$	*w1118; P{GD1354}v7762*	VDRC stock 7762		Weak	
Actn$^{RNAi\text{-}HMS00193}$	*y[1] sc[*] v[1] sev[21]; P{y[+t7.7] v[+t1.8]=TRiP.HMS00193}attP2*	BDSC stock 34874		Weak	
Actn$^{RNAi\text{-}KK102286}$	*P{KK102286}VIE-260B*	VDRC stock 110719	(Czajkowski et al., 2021)	Weak	
DAAM$^{RNAi\text{-}GD8382}$	*w1118; P{GD8382}v24885*	VDRC stock 24885		Strong	yes
DAAM$^{RNAi\text{-}HMS01978}$	*y[1] sc[*] v[1] sev[21]; P{y[+t7.7] v[+t1.8]=TRiP.HMS01978}attP2*	BDSC stock 39058		Weak	
DAAM$^{RNAi\text{-}KK102786}$	*P{KK102786}VIE-260B*	VDRC stock 103921	(Sherrard et al., 2021)	Medium	
dia$^{RNAi\text{-}KK101745}$	*P{KK101745}VIE-260B*	VDRC stock 103914	(Sherrard et al., 2021)	Strong	yes
dia$^{RNAi\text{-}HM05027}$	*y[1] v[1]; P{y[+t7.7] v[+t1.8]=TRiP.HM05027}attP2*	BDSC stock 28541		Weak	
dia$^{RNAi\text{-}HMS00308}$	*y[1] sc[*] v[1] sev[21]; P{y[+t7.7] v[+t1.8]=TRiP.HMS00308}attP2*	BDSC stock 33424		Weak	
dia$^{RNAi\text{-}HMS06017}$	*y[1] sc[*] v[1] sev[21]; P{y[+t7.7] v[+t1.8]=TRiP.HMS06017}attP40*	BDSC stock 80437		Weak	
dia$^{RNAi\text{-}GD9442}$	*w1118; P{GD9442}v20518*	VDRC stock 20518	(Sherrard et al., 2021)	Weak	
Ecad$^{RNAi\text{-}A106A1}$	*UAS-Ecad*$^{RNAi\ [A106A1]}$	(Seppa et al., 2008)	(Seppa et al., 2008)	Medium	
Ecad$^{RNAi\text{-}B107A1}$	*UAS-Ecad*$^{RNAi\ [B107A1]}$	(Seppa et al., 2008)	(Seppa et al., 2008)	Strong	yes
Ecad$^{RNAi\text{-}E206A1}$	*UAS-Ecad*$^{RNAi\ [E206A1]}$	(Seppa et al., 2008)	(Seppa et al., 2008)	Medium	
ena$^{RNAi\text{-}GD8910}$	*w1118; P{GD8910}v43056*	VDRC stock 43056		Strong	yes
ena$^{RNAi\text{-}GD8910}$	*w1118; P{GD8910}v43058/CyO*	VDRC stock 43058	(Sherrard et al., 2021)	Medium	

Development • Supplementary information

kst[RNAi-GD1472]	*w1118; P{GD1472}v37075*	VDRC stock 37075	(Deng et al., 2020)	Strong	yes
kst[RNAi-GD1472]	*w1118; P{GD1472}v37074*	VDRC stock 37074		Medium	
qua[RNAi-GD11926]	*w1118; P{GD11926}v27623*	VDRC stock 27623		Weak	
qua[RNAi-KK103765]	*P{KK103765}VIE-260B*	VDRC stock 100856		Strong	yes
w[RNAi]	*y[1] v[1]; P{y[+t7.7] v[+t1.8]=TRiP.HMS00017}attP2*	BDSC stock 33623		Strong	yes

1. Strength of knockdown in the eyes when driven by *GMR-GAL4*
2. Strong *w* knockdown, but no eye mis-patterning

Development • Supplementary information

Table S15. Antibodies utilized in this study

**Primary antibody**	**Dilution**	**Source**	**Citation**
mouse anti-Actn	1 in 50	Developmental Studies Hybridoma Bank, DSHB Cat# 2G3-3D7 RRID:AB_2721943	(Saide et al., 1989)
mouse anti-α-Spec	1 in 50	Developmental Studies Hybridoma Bank, DSHB Cat# 3A9 RRID:AB_528473	
rabbit anti-DAAM	1 in 1000	Gift from Jozsef Mihaly, University of Szeged	(Gazso-Gerhat et al., 2023)
rabbit anti-Dia	1 in 1000	Gift from Steven Wasserman, University of California San Diego	(Castrillon and Wasserman, 1994)
mouse anti-Dlg1	1 in 40	Developmental Studies Hybridoma Bank, DSHB Cat# 4F3 RRID:AB_528203	(Parnas et al., 2001)Parnas et al,2001
rat anti-Ecad	1 in 40	Developmental Studies Hybridoma Bank, DSHB Cat# DCAD2, RRID:AB_528120	(Oda et al., 1994)
mouse anti-Ena	1 in 40	Developmental Studies Hybridoma Bank, DSHB Cat# 5G2 RRID:AB_528220	(Bashaw et al., 2000)
chicken anti-GFP	1 in 10000	Abcam Ltd, Cat# 13970	
rabbit anti-Kst	1 in 500	Gift from Claire Thomas, The Pennsylvania State University	(Thomas and Kiehart, 1994)
rabbit anti-pMLC	1 in 30	Cell Signaling Technology, Cat#3671S	
mouse anti-Qua	1 in 50	Developmental Studies Hybridoma Bank, DSHB Cat# 6B9 RRID:AB_528447	(Mahajan-Miklos and Cooley, 1994)
**Secondary antibody**			
Cy™3 AffiniPure™ Goat anti-Mouse IgG (H+L)	1 in 200	Jackson ImmunoResearch Laboratories Inc. Cat# 115-165-166	
Cy™3 AffiniPure™ Goat anti-Rabbit IgG (H+L)	1 in 200	Jackson ImmunoResearch Laboratories Inc. Cat# 115-165-144	
Alexa Fluor® 647 AffiniPure™ Donkey anti-Rat IgG (H+L)	1 in 200	Jackson ImmunoResearch Laboratories Inc. Cat# 712-605-153	
Alexa Fluor® 488 AffiniPure™ Donkey Anti-Chicken IgY (IgG) (H+L)	1 in 300	Jackson ImmunoResearch Laboratories Inc. Cat# 703-545-155	

Development • Supplementary information

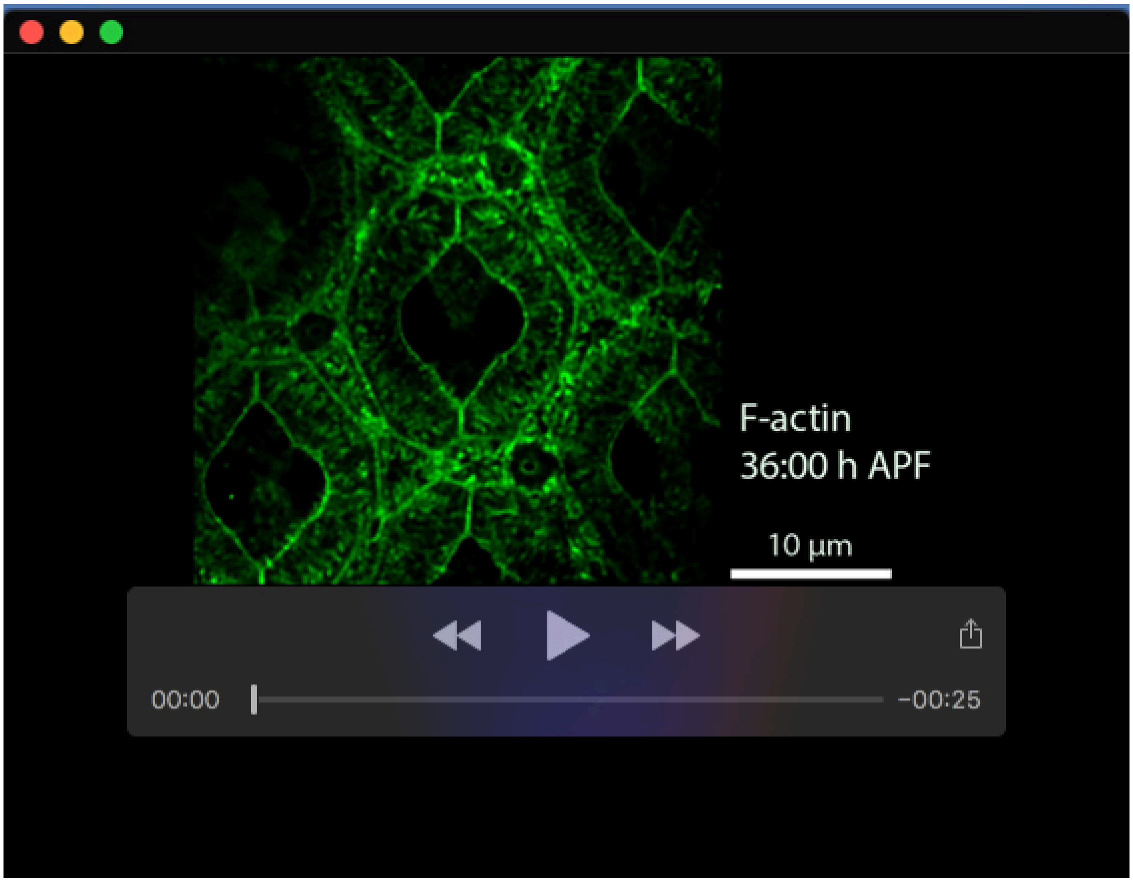

Movie 1. An ommatidium in a retina imaged from 35 to 43 h APF. Lifeact^{GFP} labels F-actin, driven by *GMR-GAL4* . Expression of Lifeact^{GFP} was inconsistent in CCs, and bristles are also not labelled in the ommatidia shown. ARAFs become increasingly dense and organized over time. Resolution of F-actin when imaged in the live eye is lower than when imaged in tissue dissected from the pupal head.

Development • Supplementary information

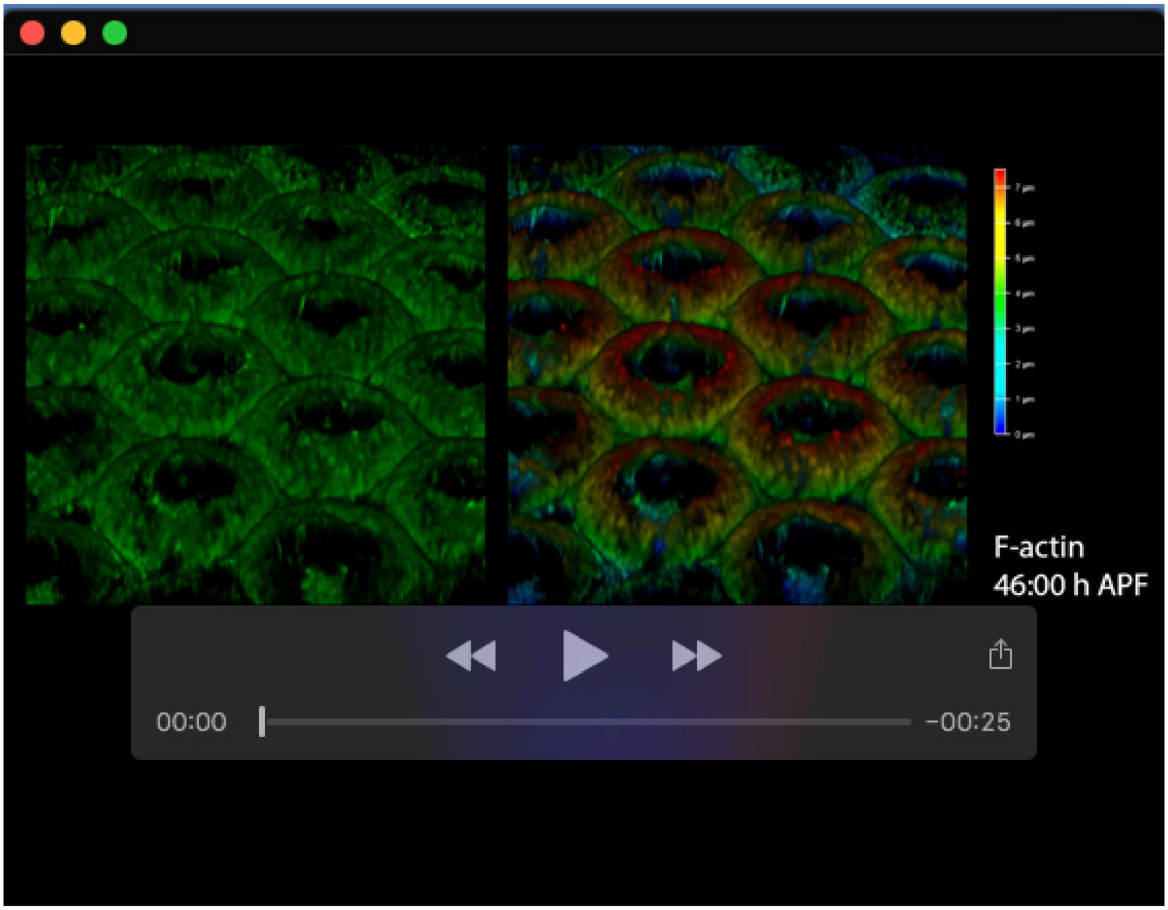

Movie 2. Small region of an eye imaged from 45 to 55 h APF. Lifeact^{GFP} labels F-actin and was not expressed in most CCs. Live-imaging data are presented as 3-D perspective projections, with the depth-coding perspectives on the right. ARAFs become increasingly dense and ommatidial doming more pronounced through the course of this movie.

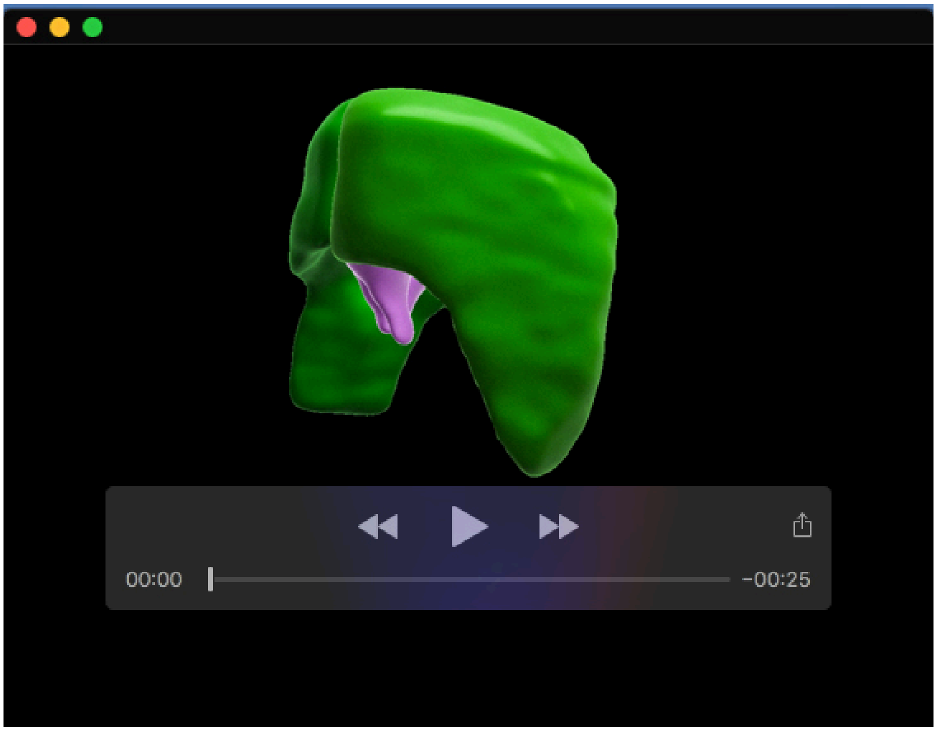

Movie 3. 3-D rendering of a pair of 1°s and the CCs of an ommatidium. 1°s (green) and CCs (pink) extend narrow projections to the base of the ommatidium that were not captured in our 3-D rendering. See also Fig.12.

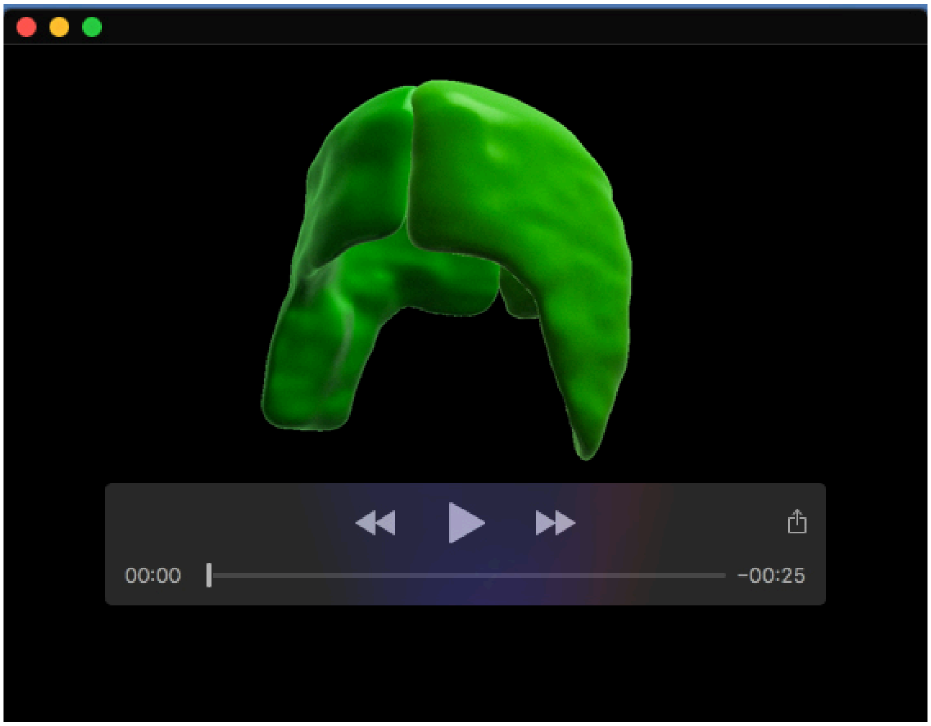

Movie 4. 3-D rendering of a pair of 1°s. The 1°s connect to each other only apically. See also Fig. 12.

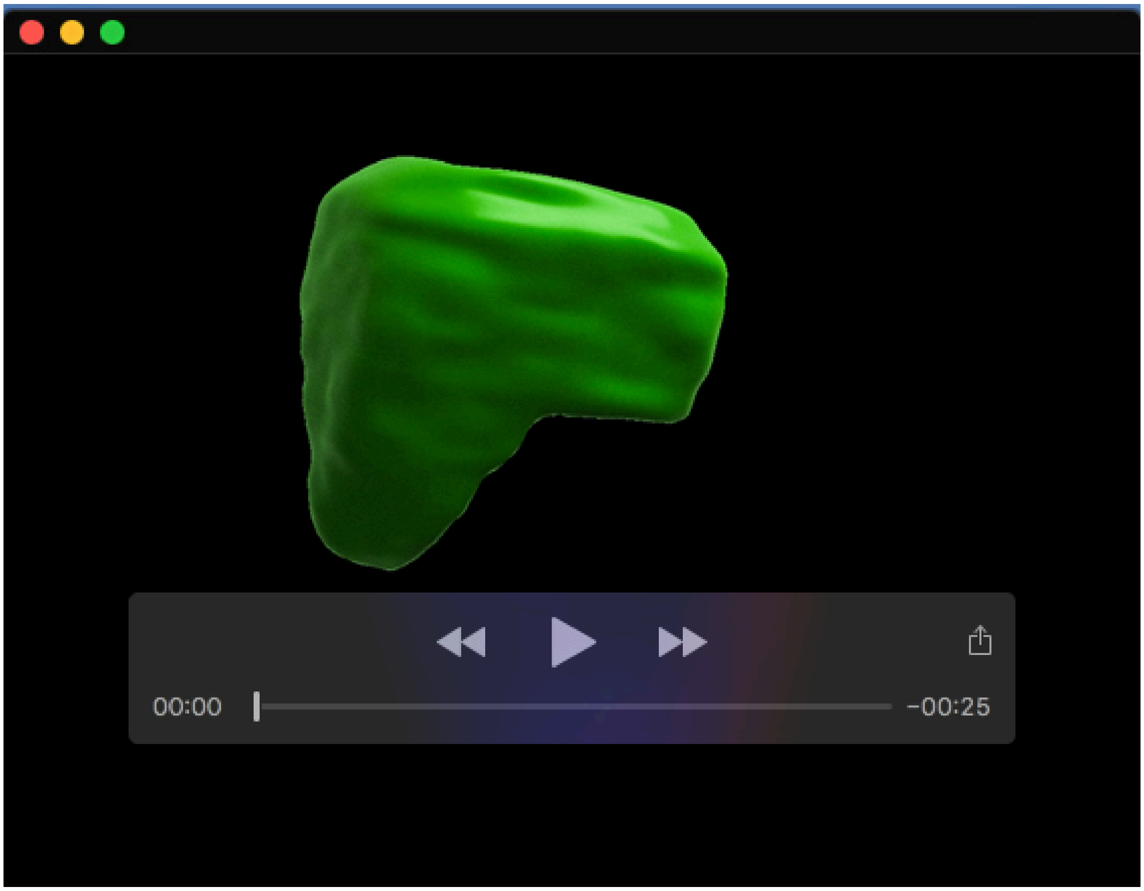

Movie 5. 3-D rendering of a single of 1°. The shape of 1° cells is similar to that of a shark tooth. See also Fig. 12.

Development • Supplementary information

Supplementary References

Bashaw, G.J., Kidd, T., Murray, D., Pawson, T. and Goodman, C.S., 2000. Repulsive axon guidance: Abelson and Enabled play opposing roles downstream of the roundabout receptor. Cell. 101, 703-15.

Castrillon, D.H. and Wasserman, S.A., 1994. Diaphanous is required for cytokinesis in Drosophila and shares domains of similarity with the products of the limb deformity gene. Development. 120, 3367-77.

Clark, K.A. and Kadrmas, J.L., 2013. Drosophila melanogaster muscle LIM protein and alpha-actinin function together to stabilize muscle cytoarchitecture: a potential role for Mlp84B in actin-crosslinking. Cytoskeleton (Hoboken). 70, 304-16.

Czajkowski, E.R., Cisneros, M., Garcia, B.S., Shen, J. and Cripps, R.M., 2021. The Drosophila CG1674 gene encodes a synaptopodin 2-like related protein that localizes to the Z-disc and is required for normal flight muscle development and function. Dev Dyn. 250, 99-110.

Deng, H., Yang, L., Wen, P., Lei, H., Blount, P. and Pan, D., 2020. Spectrin couples cell shape, cortical tension, and Hippo signaling in retinal epithelial morphogenesis. J Cell Biol. 219.

Gazso-Gerhat, G., Gombos, R., Toth, K., Kaltenecker, P., Szikora, S., Biro, J., Csapo, E., Asztalos, Z. and Mihaly, J., 2023. FRL and DAAM are required for lateral adhesion of interommatidial cells and patterning of the retinal floor. Development. 150.

Khanna, M.R., Mattie, F.J., Browder, K.C., Radyk, M.D., Crilly, S.E., Bakerink, K.J., Harper, S.L., Speicher, D.W. and Thomas, G.H., 2015. Spectrin tetramer formation is not required for viable development in Drosophila. J Biol Chem. 290, 706-15.

Mahajan-Miklos, S. and Cooley, L., 1994. The villin-like protein encoded by the Drosophila quail gene is required for actin bundle assembly during oogenesis. Cell. 78, 291-301.

Oda, H., Uemura, T., Harada, Y., Iwai, Y. and Takeichi, M., 1994. A Drosophila homolog of cadherin associated with armadillo and essential for embryonic cell-cell adhesion. Dev Biol. 165, 716-26.

Parnas, D., Haghighi, A.P., Fetter, R.D., Kim, S.W. and Goodman, C.S., 2001. Regulation of postsynaptic structure and protein localization by the Rho-type guanine nucleotide exchange factor dPix. Neuron. 32, 415-24.

Saide, J.D., Chin-Bow, S., Hogan-Sheldon, J., Busquets-Turner, L., Vigoreaux, J.O., Valgeirsdottir, K. and Pardue, M.L., 1989. Characterization of components of Z-bands in the fibrillar flight muscle of Drosophila melanogaster. J Cell Biol. 109, 2157-67.

Santa-Cruz Mateos, C., Valencia-Exposito, A., Palacios, I.M. and Martin-Bermudo, M.D., 2020. Integrins regulate epithelial cell shape by controlling the architecture and mechanical properties of basal actomyosin networks. PLoS Genet. 16, e1008717.

Seppa, M.J., Johnson, R.I., Bao, S. and Cagan, R.L., 2008. Polychaetoid controls patterning by modulating adhesion in the Drosophila pupal retina. Dev Biol. 318, 1-16.

Sherrard, K.M., Cetera, M. and Horne-Badovinac, S., 2021. DAAM mediates the assembly of long-lived, treadmilling stress fibers in collectively migrating epithelial cells in Drosophila. Elife. 10.

Thomas, C.M. and Kiehart, D.P., 1994. Beta heavy-spectrin has a restricted tissue and subcellular distribution during Drosophila embryogenesis. Development. 120, 2039-50.
